# Isolation, characterization, molecular analysis and application of bacteriophage DW-EC to control Enterotoxigenic *Escherichia coli* on various foods

**DOI:** 10.1038/s41598-021-04534-8

**Published:** 2022-01-11

**Authors:** Marta Nisita Dewanggana, Clare Evangeline, Maurita Delia Ketty, Diana Elizabeth Waturangi, Yogiara, Stella Magdalena

**Affiliations:** 1 Biotechnology Department, Faculty of Biotechnology, Atma Jaya Catholic University of Indonesia, Jenderal Sudirman 51 Street, South Jakarta, DKI Jakarta, 12930 Indonesia; 2 Food Technology Department, Faculty of Biotechnology, Atma Jaya Catholic University of Indonesia, Jakarta, Indonesia

**Keywords:** Microbiology, Molecular biology

## Abstract

Among food preservation methods, bacteriophage treatment can be a viable alternative method to overcome the drawbacks of traditional approaches. Bacteriophages are naturally occurring viruses that are highly specific to their hosts and have the capability to lyse bacterial cells, making them useful as biopreservation agents. This study aims to characterize and determine the application of bacteriophage isolated from Indonesian traditional Ready-to-Eat (RTE) food to control Enterotoxigenic *Escherichia coli* (ETEC) population in various foods. Phage DW-EC isolated from Indonesian traditional RTE food called dawet with ETEC as its host showed a positive result by the formation of plaques (clear zone) in the bacterial host lawn. Transmission electron microscopy (TEM) results also showed that DW-EC can be suspected to belong to the *Myoviridae* family. Molecular characterization and bioinformatic analysis showed that DW-EC exhibited characteristics as promising biocontrol agents in food samples. Genes related to the lytic cycle, such as lysozyme and tail fiber assembly protein, were annotated. There were also no signs of lysogenic genes among the annotation results. The resulting PHACTS data also indicated that DW-EC was leaning toward being exclusively lytic. DW-EC significantly reduced the ETEC population (*P* ≤ 0.05) in various food samples after two different incubation times (1 day and 6 days) in chicken meat (80.93%; 87.29%), fish meat (63.78%; 87.89%), cucumber (61.42%; 71.88%), tomato (56.24%; 74.51%), and lettuce (46.88%; 43.38%).

## Introduction

Enterotoxigenic *Escherichia coli* (ETEC) is one of the most prevalent foodborne pathogens that causes diarrhea and can persist in a wide variety of food-related environments due to its ability to attach, colonize, and form biofilms on these surfaces^1,2^. In Indonesia, there are more than seven million diarrheal cases that happened in 2020 with 14.5% death cases caused by diarrheal to infant and children^3^.

Current strategies to control the pathogen in food are either using physical treatments or chemical agents. However, there are some disadvantages in conventional methods, such as the loss of organoleptic compounds and the possibility of residual toxic material^4,5^. Therefore, it is important to explore an alternative treatment to control bacterial contamination in food.

Bacteriophages are naturally abundant in the environment and infect specific bacteria. Lytic bacteriophages are frequently used for inactivation and control of foodborne pathogens^6^. Several bacteriophages have been applied as food preservatives and categorized as generally recognized as safe (GRAS) by the Food and Drug Administration (FDA); therefore, this method is considered promising for application as a biopreservative for preventing foodborne pathogens^6^.

Bacteriophage DW-EC is a phage isolated from dawet, an Indonesian traditional Ready-To-Eat (RTE) food with ETEC as its host. The objectives of this research were to isolate bacteriophages, characterize, determine the application of several foods to control ETEC and analyze the genotype properties of bacteriophage DW-EC.

## Results

### Bacteriophage isolation from food sample, titer determination and characterization

DW-EC was isolated from Indonesian traditional Ready-to-Eat (RTE) food called dawet. It showed a positive result for ETEC as host bacteria, which was indicated by the plaque formed in the agar overlay assay. DW-EC had circular and clear plaques with a diameter of approximately 0.8 mm and titer of 1.86 ± 3.21 × 10^8^ PFU/mL. The titer determination was tested in triplicate. (Supplementary Table S1).

### Host spectrum determination and efficiency of plating (EOP)

Host range spectrum determination was done to check the ability of phage DW-EC to lyse other bacteria besides its own host bacteria. For host range spectrum determination, DW-EC can also infect EHEC and EPEC but not *Salmonella typhimurium, Escherichia coli* ATCC 25922, and *Vibrio cholerae*. EOP was done after the host spectrum determination to see the efficiency of DW-EC to lyse other bacteria besides its own host bacteria. DW-EC was also found to be highly effective against EHEC and EPEC, with an EOP of more than 0.5 compared to its original host (ETEC) (Supplementary Table S2 & Supplementary Table S3).

### Bacteriophage storage stability in 4 °C

DW-EC storage stability was tested at a temperature of 4 °C for a duration of 21 weeks. The titer determination of phage before storage was 4.47 × 10^9^ PFU/mL, while after 21 weeks, the final titer was 2.805 × 10^9^ PFU/mL. It showed a 37.25% bacteriophage activity reduction.

### Bacteriophage morphological analysis with transmission electron microscopy

**Figure 1:**
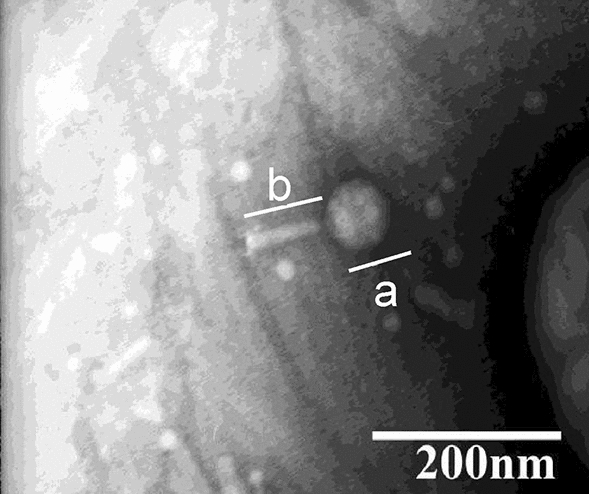
Bacteriophage DW-EC morphology by TEM at 40,000 magnifications with icosahedral head length (**a**) of approximately 75 nm and 85 nm for the tail length (**b**).

DW-EC was approximately 158–160 nm in size from determination using transmission electron microscopy. It showed an icosahedral head that connected to a tail by a short neck, with icosahedral head length (a) of approximately 75 nm and 85 nm for the tail length (b) (Fig. 1).

### Bacteriophage genomic isolation and next generation sequencing

DW-EC genomic material was isolated and subjected to next-generation sequencing (NGS). NGS results yielded DNA 151.876 bp in length with 39.07% GC content. Bacteriophage genome sequence has been submitted to GenBank with the accession number OL739525.

### Bacteriophage annotation, and phylogenetic analysis of tail fiber protein

DW-EC was annotated with multiPhATE2 using various databases. Among the results, genes associated with phage structures, cell lysis, assemblies, and packaging during the end of the lytic cycle were annotated (Table 1). Complete annotation can be viewed on Supplementary Table S4.

**Table 1:** Notable DW-EC annotation results.

CDs	Annotation	Source of organism	Association
DW-EC-86	Putative T4-like lysozyme (EC 3.2.1.17)	*Escherichia* phage phAPEC8	Cell lysis
DW-EC-146	Phage tail sheath protein; baseplate wedge subunit	Uncultured Mediterranean phage uvMED-GF-U-MedDCM-OCT-S28-C30; *Edwardsiella* phage PEi26	Structural
DW-EC-149	Putative terminase	*Escherichia* phage phAPEC8	Packaging
DW-EC-153	Putative head stabilization/decoration protein	*Escherichia* phage phAPEC8	Structural
DW-EC-154	Putative major head protein	*Escherichia* phage phAPEC8	Structural
DW-EC-161	Putative tail tube	*Enterobacteria* phage ECGD1	Structural
DW-EC-174	Tail fiber protein; endo-*N*-acetylneuraminidase	*Escherichia* phage phAPEC8	Structural
DW-EC-175	Tail spike protein; endo-*N*-acetylneuraminidase	*Salmonella* phage FSL SP-076; *Escherichia* phage vB_EcoM_CBA120	Structural
DW-EC-179	Putative gpH domain protein	*Escherichia* phage phAPEC8	Assembly
DW-EC-180	Putative tail fiber assembly protein	*Escherichia* phage phAPEC8	Assembly
DW-EC-183	Putative phage tail fiber protein	*Escherichia* phage phAPEC8	Structural
DW-EC-239	Terminase; gp5; gp74	*Listeria* phage LMTA-94; *Listeria* virus P100	Packaging
DW-EC-277	Phage minor capsid protein	Bacteriophage SPP1	Structural
DW-EC-302	Terminase small subunit	*Geobacillus* phage GBSV1	Packaging

**Figure 2:**
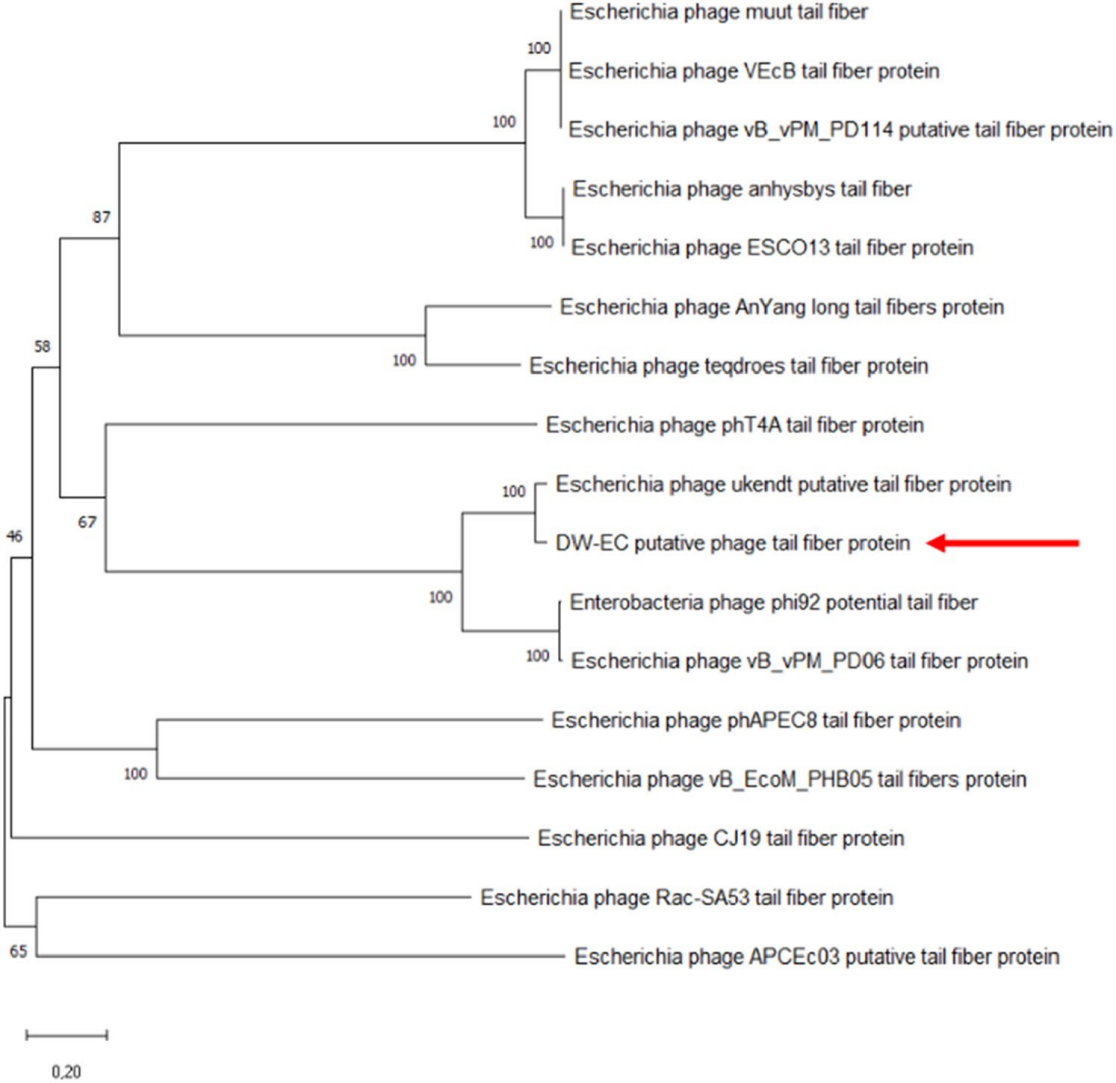
Unrooted phylogenetic tree of DW-EC tail fiber protein and other related phages taken from NCBI database (100 bootstrap, 20% homology). DW-EC is indicated by arrows (←).

A phylogenetic tree based on phage tail fiber protein sequences was constructed for DW-EC. Sixteen additional tail fiber protein sequences were obtained from NCBI databases, those with *E. coli* of various variances as its host. The DW-EC (Fig. 2) tail fiber protein was shown to have the closest similarity to the *Escherichia* phage ukendt tail fiber protein.

**Figure 3:**
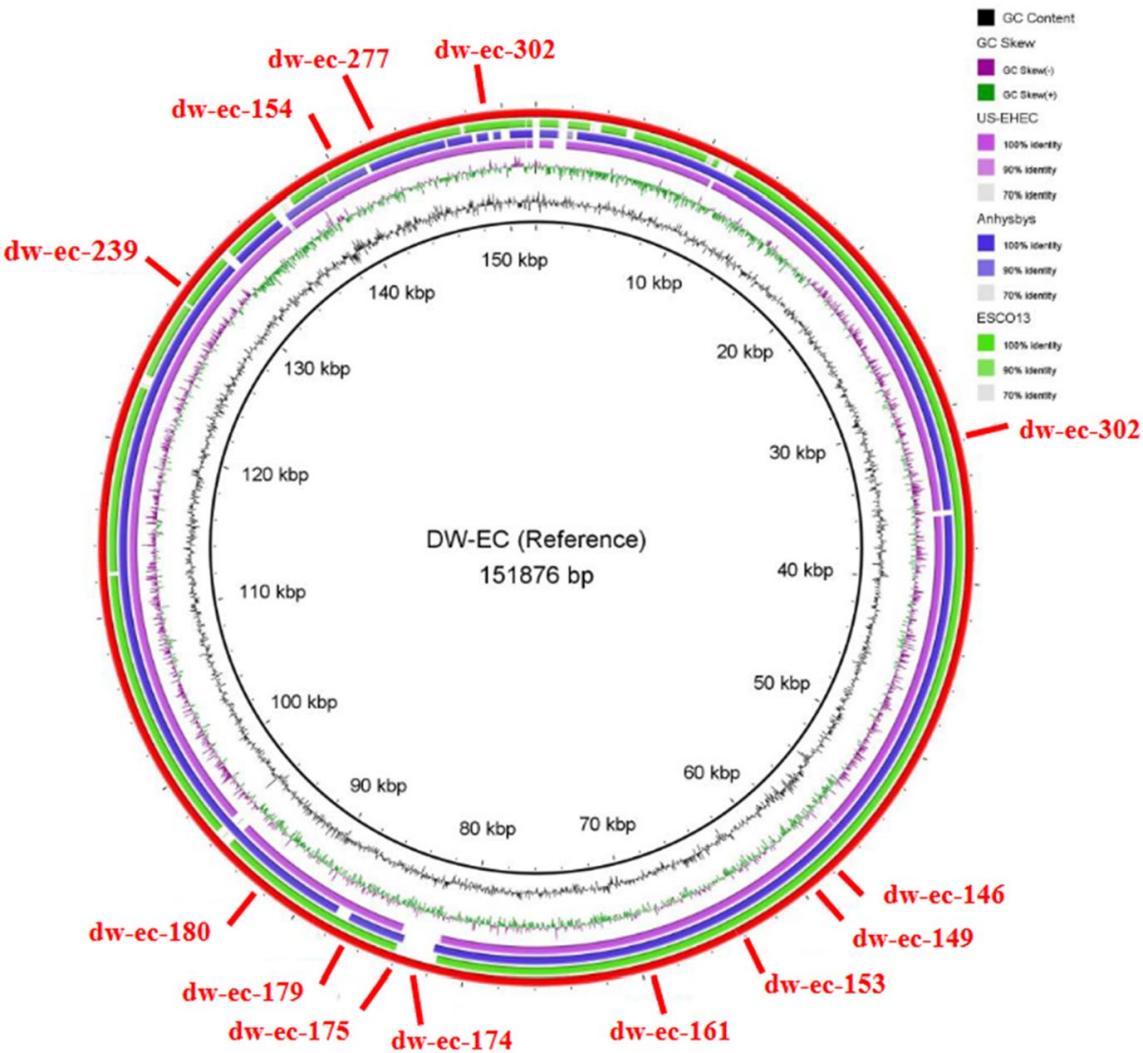
Comparative genomic analysis of DW-EC and the other bacteriophages. The inner circle is the DW-EC genome as a reference. Others included US-EHEC (purple), anhysbys (blue), ESCO13 (green), and DW-EC annotation (red). Colored rings showed similarity between each phage.

BLAST analysis on DW-EC showed the highest similarity to *Escherichia* phage anhysbys (NCBI accession No. NC_052656.1). For BRIG, additional *Escherichia* phages US-EHEC (unpublished data) and ESCO13 (NCBI accession No. NC_047770.1) were selected along with anhysbys to serve as a comparison genome (Fig. 3).

PHACTS analysis on DW-EC revealed that while it could not confidently declare samples as lytic phages, it led toward being one. The average probability produced by PHACTS for DW-EC is 0.507 with a standard deviation of 0.036.

CARD analysis performed with perfect and strict parameters yielded zero results, and it was changed to loose hits to accommodate. Loose hits parameter yieled LRA-5 as the highest result with 80 in best identity value.

### Bacteriophage application on food samples

The food samples were stored in two different incubation times which were 1 day and 6 days at 4 °C storage temperature, ETEC was reduced significantly in chicken meat samples (80.93%; 87.29%), fish meat (63.78%; 87.89%), cucumber (61.42%; 71.88%), tomato (56.24%; 74.51%), and lettuce (46.88%; 43.38%) (Supplementary Table S5). All of the assays were done in triplicate.

## Discussions

Morphological characterization based on TEM showed that DW-EC (Fig. 1) belongs to the *Myoviridae* family because of its larger head size with a long, rigid and contractile tail^7^. Clear and circular plaques indicated phage DW-EC as a lytic bacteriophage^8^.

DW-EC had a highly specific host range, as it only infects several strains in the same species. Phages can determine their host cell bacteria by binding or adsorbing into the surface of the bacterial cell wall, which is called a receptor. The binding between them is influenced by several factors that can affect the attachment between phages and bacteria, including pili, capsules, teichoic acid, lipopolysaccharides (LPS) and surface proteins in bacterial cells^9^.

Phage DW-EC had a positive result with EHEC and EPEC in host range determination assay which led for the Efficiency of Plating (EOP) to be done against EHEC and EPEC for further analysis. The EOP results can be classified into four classes: high efficiency with EOP 0.5 to 1.0, moderate efficiency with EOP 0.2 to < 0.5, low efficiency with EOP 0.001 to < 0.2, and inefficient with EOP ≤ 0.001^6^. DW-EC was highly efficient against EPEC and EHEC, which made them have the potential to become host cells of DW-EC because of their high EOP values.

Storage of the phage at 4 °C showed a slight bacteriophage activity reduction (37.25%). Fortier and Moineau^10^ declared that it was better to store phage by lyophilization compared with refrigeration because it has a higher heat stability and is resistant to drying. Good storage conditions of the phage are dependent on the phage type because phage characteristics are influenced by their origin-isolated place^10^.

DW-EC, upon subjecting each genome to NGS analysis, was found to be composed of 151.876 bp with 39.07% GC content. Annotations using multiPhATE2 were able to annotate CDs that produce proteins necessary for the end of a lytic cycle or served as structural proteins (Table 1). It was also noted that among successfully annotated CDs, genes associated with the lysogenic life cycle, such as integrase and excisionase, could not be found on the resulting annotations.

The phage genome-packaging component itself consists of portal protein, small terminase and large terminase. Small terminases can be annotated using multiPhATE2 (Table 1). Small terminases are used to initiate genome packaging and regulate large terminase functions. Meanwhile, the function of a large terminase is to cleave concatenated DNA molecules to initiate packaging mechanisms and again after packaging^11^.

Assembly for *Myoviridae* phages is performed separately for the head, tail, and long tail fibers before joining to form a mature phage. Both tail fiber assembly (Tfa) and gpH were involved in tail assembly. Tfa is a family of proteins that function as chaperones for folding phage fibers and determining host range specificity on the assembled phage. Tape measures protein gpH to determine the length of the phage tail^12,13^.

Putative T4-like lysozyme is a hydrolytic enzyme used to cleave peptidoglycan bonds. It is produced during the late stage of the lytic cycle when assembled phages are ready to be released to the environment. Lysin possesses two main domains, the N-terminus and C-terminus. The N-terminus functions as a catalytic domain, while the C-terminus serves as a binding domain that targets and binds to specific ligands^14^.

Tail fibers function as receptor binding proteins (RBPs) in many bacteriophages. RBP plays a role in phage host recognition and its interaction with other phages of the same host. For T4-like phages, the C-terminal and N-terminal regions of tail fibers are important to determine receptor specificity as well as host range^15,16^. The DW-EC tail fiber protein was shown to be the closest to *Escherichia* phage ukendt with *E. coli* K-12 MG1655 as its host^17^. Similar genetic make-up might contribute to different phages having the same host range. It is beneficial to study and observe a variety of tail fiber genes to expand knowledge of the host range used in phage cocktails^18^.

Resulting annotations and BRIG analysis showed no lysogenic and virulence genes on DW-EC. Lysogenic bacteriophages utilize integrase and excisionase, encoded by *int* and *xis*, to bind its DNA to the host’s^19–21^.

Analysis using the CARD database was done to determine whether samples carry over ARGs from the host or not. A temperate phage has a higher probability of carrying host genes. The possibility of bacteriophages carrying over ARGs is possible but rare. It was suggested that up to 1000-fold uncommon for phages to transfer ARGs via transduction compared to other means^22^.

Initial analysis using the CARD was performed using perfect and strict hit-only parameters. However, this run yielded no results, which might indicate no ARGs present on DW-EC. Another analysis was then conducted with the loose hit parameter, including hits with less than 95% homology matches. The highest match for DW-EC in the CARD is LRA-5, with 80 in the best identity value. LRA-5 is a class A β-lactamase sequence found in Alaskan soil. It was reported that *E. coli* metagenomic *E. coli* containing LRA-5 is resistant to the cephalosporin drug class. β-lactamase works by hydrolyzing the β-lactam ring, ultimately rendering it inactive^23^. While ARGs were found during CARD analysis with loose hit parameters, it was still possible to rule out ARGs being present in the genomes. Another study found that some proteins might be mistakenly labeled as ARGs when using CARD. This finding is common with phage genomes containing many leftover DNA molecules from host cells. It was also suggested that the same study only use conservative parameters when using in silico analysis to achieve the best possible matches. This implies that only perfect and strict hit results are eligible to be included^22^.

**Figure 4:**
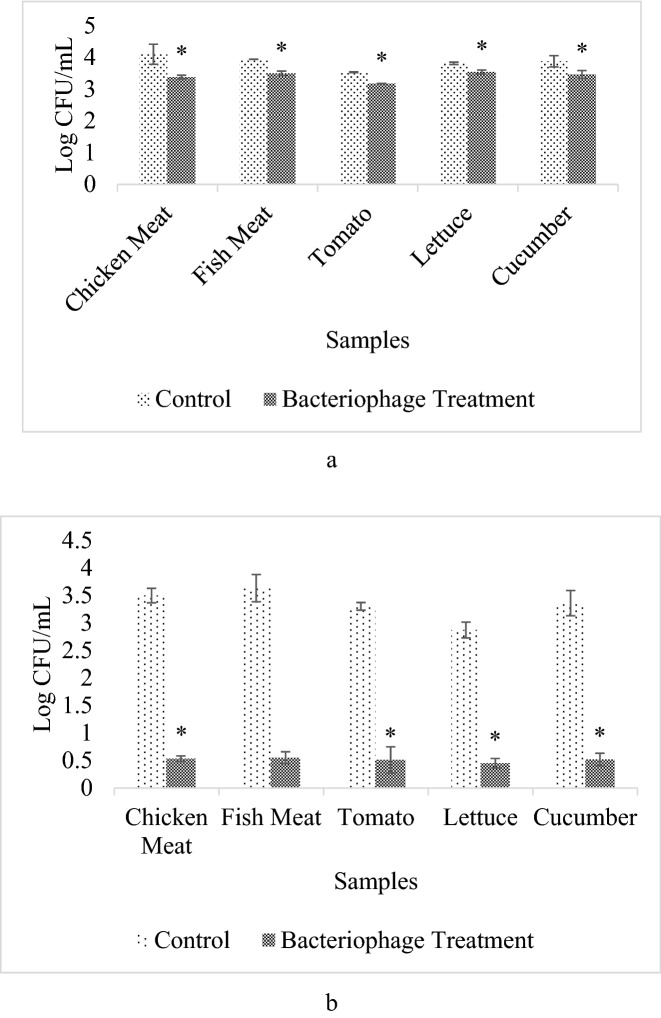
Bacteriophage DW-EC application on various food samples at 4 °C. With different time of incubation, which were for 1 day (**a**) and 6 days (**b**). “*”: Significant difference between the control and the treatment with *P* ≤ 0.05.

DW-EC was applied to various types of foods to determine its capability to lyse ETEC. Based on the results shown (Fig. 4), the DW-EC bacteriophage significantly reduced ETEC in low-temperature storage (4 °C) after 1 day and 6 days of incubation, except for fish meat in 6 days incubation. Low-temperature storage was selected because it was considered the common temperature used to store raw meats, fruits, vegetables, and pasteurized milk fresh for a few days.

DW-EC adsorption to reach the bacterial host cell is affected by the various food matrices^15,24^. The structure and chemical composition of different food items can also affect DW-EC activity by allowing the food to resist the penetration of the phage into the food matrix, causing the phage to remain on the surface of the food, where they may become desiccated^25^. For example, using solid samples with uneven surfaces such as chicken and fish meat. These matrices limit the distribution of phage particles to reach all the target bacterial cells due to their matrices shield the bacterial cells^24,26^. The food matrix for liquid sample such as milk should not appear to be a problem, because suspended phage particles can diffuse almost freely, while for the solid samples with even surfaces such as fruits and vegetables, the total surface area and its ability to absorb the phage suspension are also the critical parameter of the phage particles distribution and its ability to diffuse^24,26^.

The DW-EC adsorption and diffusion rate to reach the bacterial target cell may also be affected by extrinsic factors, such as inadequate nutrition, which might affect bacterial growth, pH, temperature, and water content in the samples. High phage titer or bacterial concentrations, which are used, also contribute to bacteriophage adsorption, diffusion, and distribution to reach and lyse bacterial target cells^27^.

## Methods

### Bacterial strains

Enterotoxigenic *E. coli* (ETEC), Enteropathogenic *E. coli* (EPEC), Enterohaemorrhagic *E. coli* (EHEC), *E. coli* ATCC 25922, *Salmonella typhimurium*, and *Vibrio cholerae* were obtained from the Laboratory of Food Microbiology of Faculty of Biotechnology of Atma Jaya Catholic University of Indonesia. Hosts were grown in Luria Bertani (LB) broth to mid-log phase at 37 °C and 120 rpm for 6–8 h before use (OD_600_ = 0.132/10^8^ CFU/mL). For storage of short-term inoculums, LB agar [2% (w/v) agar] was used, grown at 37 °C overnight, and then stored in a refrigerator at 4 °C^25^.

### Bacteriophage isolation and purification

Dawet as Indonesian Ready-to-Eat (RTE) foods were sold in street and traditional markets in Tangerang, Indonesia. The ETEC strain was used as the host for isolation and characterization of bacteriophages. Dawet samples were suspended in 1:10 (w/v) salt-magnesium (SM) buffer [0.1 M NaCl, 8 mM MgSO_4_·7H_2_O, 50 mM Tris–Cl pH 7.5, 0.01% gelatin (w/v)] ratio and crushed using a stomacher (Interscience, St Nom, France) for 1 min. The suspension obtained was then mixed with the bacterial host at a ratio of 9:1 (v/v), MgSO_4_ and CaCl_2_ at 5 mM to increase the binding between the phage and the host bacteria and then incubated overnight at 37 °C and 120 rpm. Then, the sample was removed into a 2 mL sterile microtube, centrifuged at 10,000 × g for 10 min to separate the bacteriophage from its host cell, and filtered using a 0.2 µm pore-size microfilter (Axiva, Kundli, India) for phage purification to obtain a bacteriophage lysate^15,22^. The bacteriophage lysate was kept in ¼-strength Ringer Solution (OXOID) at a 1:1 (v/v) ratio. The solution was stored at 4 °C as a working solution^28^.

### Bacteriophage titer determination, enrichment, and characterization

Phage lysate was diluted with SM buffer by a series of tenfold dilutions. Titer determination was performed with an agar overlay assay, in which 250 µL of diluted bacteriophage filtrate, 250 µL of mid-log phase bacterial strain (OD_600_ = 0.132/10^8^ CFU/mL), 50 µL of 10 mM CaCl_2_, and 10 µL of 10 mM MgSO_4_ were mixed^28^. The mixture was vortexed and incubated for 20 min at 28 °C. After incubation, the mixture was mixed with 5 mL of molten LB agar [0.6% (w/v) agar] and then poured onto LB agar. The plate was incubated at 37 °C overnight, and plaque formation was observed. The visible plaques were counted on the appropriate dilutions, giving between 3 and 300 plaques, and the titer was calculated in plaques forming units per millimeter (PFU/mL)^25,29^. The morphology and diameter of the plaques were measured manually^30^.

For phage enrichment, the plaque obtained from the previous step can be retrieved (along with molten LB agar) by a loop or sucked using a micropipette, mixed with LB broth with 250 µL of mid-log phase ETEC strain, 50 µL of 10 mM CaCl_2_, and 10 µL of 10 mM MgSO_4_, and then incubated at 37 °C overnight at 120 rpm. The procedure was similar between bacteriophage isolation and titer determination assays^25,29,30^. The titer determination of phage was tested in triplicate.

### Host spectrum determination and efficiency of plating (EOP)

The phage that was isolated was tested against several bacterial species and strains, including ETEC, EHEC, EPEC, *E. coli* ATCC 25922, *Salmonella typhimurium*, and *Vibrio cholerae.* The test was performed using an agar overlay assay as described for titer determination, and a positive result was indicated by plaque formation between the host bacterial lawn after incubation at 37 °C for 18–24 h^31^. Bacteriophages that were able to lyse the bacterial strains in host range determination were subjected to the efficiency of plating (EOP) assay.

ETEC was used as the host cell for the bacteriophage, while other bacteria were used as other target bacteria. The phage lysate was diluted to 10^−7^ with sterilized SM buffer. Dilution from 10^−4^ to 10^−7^ was used, pipetted to 250 µL and mixed alongside 250 µL of mid-log phase bacterial strain (OD_600_ = 0.132/10^8^ CFU/mL), 50 µL of 10 mM CaCl_2_, and 10 µL of 10 mM MgSO_4_^32^. The mixture was vortexed and incubated for 20 min at 28 °C. After incubation, the mixture was mixed with 5 mL of molten LB agar [0.6% (w/v) agar] and then poured onto LB agar. The plate was incubated at 37 °C overnight, and plaque formation was observed. The visible plaques were counted on the appropriate dilutions, giving between 3 and 300 plaques. When the 10^−4^ dilution did not result in any plaques, a lower dilution was tried afterward to verify the lower EOP. Eventually, EOP was calculated by dividing the average PFU on target bacteria by the average PFU on host bacteria^33^.

### Bacteriophage storage stability in 4 °C

The isolated phage diluted in Ringer solution (stocked phage 10^9^ PFU/mL) was stored in a chiller (4 °C) for 21 weeks. The titer determination before storage and after a certain period of storage was tested using an agar overlay assay and then compared to determine the viability of the phage^9^.

### Bacteriophage morphological analysis with transmission electron microscopy (TEM)

Bacteriophage identification and classification were conducted using transmission electron microscopy (TEM) at the Eijkman Institute for Molecular Biology. Bacteriophage isolate was dropped into the grids (carbon film copper grids) and negatively stained with 2% uranyl acetate. The stained specimens were dried using filter paper and observed using a JEM-1010 transmission electron microscope (JEOL, Tokyo, Japan) at 40,000× magnification. Phage identification and classification were conducted according to the International Committee on Taxonomy of Viruses guidelines^9^.

### Bacteriophage DNA extraction

Bacteriophage DNA isolation was performed by adding 5 μL of DNaseI to 1 mL of purified bacteriophage. The solution was incubated at 37 °C for 30 min. Nucleic acid was extracted by adding 6 μL of EDTA 0.05 M, 10 μL of 1% sodium dodecyl sulfate (SDS) and 6 μL of proteinase K (10 mg/mL). The mixture was incubated at 37 °C for 1 h. After incubation, 600 μL of phenol–chloroform–isoamyl alcohol solution (25:24:1) was added to remove unwanted materials, and the solution was centrifuged at 2655 g for 5 min. The upper phase was taken into a new microtube, mixed with 500 μL of chloroform-isoamyl alcohol solution (24:1), and centrifuged at 2655 × g for 5 min. The upper phase was taken into a new microtube. A 3 M sodium acetate pH 5.2 (1:10) solution followed by isopropyl alcohol (1:1) was added to the mixture. Isopropyl alcohol was added to precipitate the DNA. The mixture was then incubated in ice for 15 min. After incubation, the suspension was centrifuged at 17,949 × g for 10 min, and the supernatant was removed. Approximately 700 μL of 70% ethanol was added to the pellet, and the mixture was centrifuged again at 17,949 × g for 10 min. The supernatant was removed, and the pellet was dried. Fifty microliters of nuclease-free water (NFW) solution was added to the pellet for DNA storage at 4 °C^34^.

### Next generation sequencing (NGS)

DNA sequences obtained from bacteriophage genomic isolation were sent to PT Genetika Science Indonesia for NGS using Oxford Nanopore Technologies (MinKNOW 20.06.9). Base calling was performed using Guppy 4.0.11 high accuracy mode. Raw NGS data were filtered using Filtlong v.0.2.0^35^. De novo assembly was performed with Flye v.2.8.3^35,36^ using default parameters for single genome assembly. Medaka 1.2.0^37^ was used to polish the assembled genome.

### Bacteriophage annotation, and phylogenetic analysis of tail fiber protein

Genome annotations were carried out with multiPhATE2^38^ using default and supporting databases. A phylogenetic tree was also constructed using MEGAX^39^. Multiple sequence alignment was performed using ClustalW, and a phylogenetic tree was constructed with the neighbor-joining method^18^.

BLAST analysis was carried out to determine that the similarity DW-EC most resembles^40^. Two bacteriophages from the NCBI database were chosen to be compared with DW-EC using Blast Ring Image Generator (BRIG)^41^. BRIG analysis was also performed on lysogenic genes (*xis* and *int*), both of which were taken from the NCBI database.

Further analysis was done using CARD^42^ to study the presence of antimicrobial resistance genes that might be present in these genomes of the samples. PHACTS^43^ was also performed to determine life cycle bacteriophages.

### Bacteriophage application on food samples

Raw chicken meat, raw fish meat, fresh lettuce, fresh cucumber, fresh tomato, and pasteurized milk were obtained from the local supermarket and were used as food samples. Raw chicken meat and raw fish meat were cut into pieces (1 cm × 1 cm) and placed in 50 mL Falcon tubes (Corning^®^) of approximately 1 g for each tube. Pasteurized milk was placed in 15 mL Falcon tubes (Corning^®^) with a volume of 1 mL for each tube. These tubes with a sample were sterilized by autoclaving for 15 min at 121°C^5^. For fresh lettuce, fresh tomato and fresh cucumber were rinsed with clean water, swabbed with 96% alcohol on their surfaces and cut into pieces (1 cm × 1 cm) with a sterilized knife/scalpel. These samples were exposed to UV light from laminar airflow (ESCO) for approximately 40 min to ensure the killing of any possible natural microbiota. After that, each piece of the sample was placed into 50 mL Falcon tubes (Corning^®^)^32,44^. After sterilization, all of the samples were inoculated with 100 µL of mid-log phase ETEC strain (10^6^ CFU/mL). These tubes were incubated for 30 min at 28 °C. Subsequently, 100 µL of phage lysate with a multiplicity of infection (MOI) of 100 was added^45,46^.

Samples were incubated at 4 °C for 1 day and 6 days. Following the incubation, 10 mL of SM buffer (0.05 M Tris–HCl, pH 7.5, 0.1 M NaCl, 0.008 M MgSO_4_, 0.01% gelatin) was added to the samples^47^. The tubes were vortexed and serially diluted up to 10^−3^. Each dilution was spread onto LB agar, and the plates were incubated at 37 °C overnight. The viable bacterial count was determined by colony forming units per milliliter (CFU/mL)^6^. For the positive control, each tube was inoculated with only the ETEC strain, and for the negative control, each tube was inoculated with only phage lysate.

### Statistical analysis

All data are presented as the means and standard error. For statistical analysis, one-way ANOVA (SPSS Inc. IBM corporation) followed by Tukey’s B test with the level of difference defined at *P* ≤ 0.05. Different letters in each column indicate significant differences from other samples in that column. For each sample, pairing (control-treatment pairing) in bacteriophage application on food samples was checked for its significant reduction by using a paired-samples T-test with the level of difference defined at *P* ≤ 0.05^47^.

## Conclusions

DW-EC was successfully isolated from dawet with ETEC as the host cell. DW-EC was highly effective against EHEC and EPEC as well as its host. It also had the capability to significantly reduce ETEC with two different incubation times (1 day and 6 days) at 4 °C storage temperature. Phage DW-EC can be suspected to be a *Myoviridae* family by TEM based on its morphology. Molecular and bioinformatic analyses also revealed that there were no signs of lysogenic or virulence genes among the results. PHACTS data also indicated that DW-EC was leaning toward being exclusively lytic. Therefore, DW-EC was found to exhibit characteristics as a promising biocontrol agent in food samples.

## Supplementary Information

Supplementary Information.

## Data Availability

All data generated or analyzed during this study are included in this published article.
