# Supplementary material for: Isolation, characterization, molecular analysis and application of bacteriophage DW-EC to control Enterotoxigenic Escherichia coli on various foods
Source: Sci Rep. 2022 Jan 11;12:495. doi: 10.1038/s41598-021-04534-8 (PMC8752677; doi:10.1038/s41598-021-04534-8)
Supplement: Supplementary file 1 — Supplementary Information. [file 41598_2021_4534_MOESM1_ESM.docx]

Supplementary Table S1 Bacteriophage Isolated from Dawet (DW-EC) Characterization and Titer Determination

| **Phage** | **Host Cell** | **Morphology of Plaque** | **Diameter of Plaque (mm)** | **Titer (PFU / mL)** |
| --- | --- | --- | --- | --- |
| DW-EC | ETEC | Circle and clear | ± 0.8 | 1.86 ± 3.21 x 10^8^ |

Supplementary Table S2 Spectrum Host Cell Bacteria

| **Bacteriophage** | **Isolation** | **Spectrum Host Cell Bacteria** | | | | | |
| --- | --- | --- | --- | --- | --- | --- | --- |
|  |  | **ETEC** | **EHEC** | **EPEC** | ***E. coli* ATCC 25922** | ***S. typhimurium*** | ***V. cholerae*** |
| DW-EC | ETEC | + | + | + | - | - | - |

*- means the negative result (no plaque)

Supplementary Table S3 Bacteriophage DW-EC Efficiency of Plating

| **Bacteriophage** | **Efficiency of Plating** | | | |
| --- | --- | --- | --- | --- |
|  | **ETEC** | **EHEC** | **EPEC** | ***E. coli* ATCC 25922** |
| DW-EC | **1.00** | 2.30 | 0.98 | - |

*- means the negative result (not be counted) ; The plating on the original strain of isolation (EOP = 1.0) is marked in bold

Supplementary Table S4 DW-EC full annotations

| CDs | | | Annotation |
| --- | --- | --- | --- |
| DW-EC-1 | 2 | 127 |  |
| DW-EC-2 | 300 | 449 |  |
| DW-EC-3 | 479 | 616 | Putative exodeoxyribonuclease; *Sulfitobacter* phage pcb2047-A exodeoxyribonuclease; *Sulfitobacter* phage pcb2047-C \| Protein TIC 214; Pelargonium hortorum \| exodeoxyribonuclease; *Sulfitobacter* phage pcb2047-C putative exodeoxyribonuclease; *Sulfitobacter* phage pcb2047-A |
| DW-EC-4 | 662 | 793 |  |
| DW-EC-5 | 825 | 1169 | DNHOGCFM_00017 hypothetical protein \| hypothetical protein phAPEC8_0018; *Escherichia* phage phAPEC8 \| HFBDACEP_00001 hypothetical protein \| JOODPJME_00217 hypothetical protein \| IFPLOHOB_00001 hypothetical protein \| hypothetical protein; *Escherichia* phage phAPEC8 |
| DW-EC-6 | 1159 | 1557 |  |
| DW-EC-7 | 1533 | 1898 | OAGBNOCD_00242 hypothetical protein |
| DW-EC-8 | 1958 | 2167 | HLAHOEIE_00049 hypothetical protein \| BDMKCPGI_00002 hypothetical protein \| IFPLOHOB_00003 hypothetical protein \| DNHOGCFM_00019 hypothetical protein \| hypothetical protein phAPEC8_0020; *Escherichia* phage phAPEC8 \| hypothetical protein; *Escherichia* phage phAPEC8 \| hypothetical protein phAPEC8_0020; *Escherichia* phage phAPEC8 |
| DW-EC-9 | 2160 | 2390 | Hypothetical protein phAPEC8_0021; *Escherichia* phage phAPEC8 \| JIHLJMCN_00003 hypothetical protein \| BDMKCPGI_00003 hypothetical protein \| DNHOGCFM_00020 hypothetical protein \| JOODPJME_00214 hypothetical protein \| hypothetical protein; *Escherichia* phage phAPEC8 \| hypothetical protein phAPEC8_0021; *Escherichia* phage phAPEC8 |
| DW-EC-10 | 2403 | 2627 | BDMKCPGI_00004 hypothetical protein \| IFPLOHOB_00005 hypothetical protein \| JOODPJME_00213 hypothetical proteinjihljmcn_00004 hypothetical protein \| OAGBNOCD_00245 hypothetical protein \| hypothetical protein; *Escherichia* phage phAPEC8 |
| DW-EC-11 | 2639 | 3100 | HLAHOEIE_00051 hypothetical protein \| HFBDACEP_00006 hypothetical protein \| JOODPJME_00212 hypothetical protein \| JIHLJMCN_00005 hypothetical protein \| OAGBNOCD_00246 hypothetical protein |
| DW-EC-12 | 3217 | 3735 | HLAHOEIE_00053 hypothetical protein |
| DW-EC-13 | 3741 | 4196 | HLAHOEIE_00054 hypothetical protein \| FBDACEP_00007 hypothetical protein \| OAGBNOCD_00248 hypothetical protein \| hypothetical protein; *Escherichia* phage phAPEC8 |
| DW-EC-14 | 4209 | 4646 | HLAHOEIE_00055 hypothetical protein \| DNHOGCFM_00024 hypothetical protein \| hypothetical protein phAPEC8_0025; *Escherichia* phage phAPEC8 \| JOODPJME_00210 hypothetical protein \| BDMKCPGI_00007 hypothetical protein \| hypothetical protein; *Escherichia* phage phAPEC8 , complete genome |
| DW-EC-15 | 4674 | 4973 | JOODPJME_00209 hypothetical protein \| JIHLJMCN_00009 hypothetical protein \| HLAHOEIE_00056 hypothetical protein \| BDMKCPGI_00008 hypothetical protein \| HFBDACEP_00010 hypothetical protein \| hypothetical protein; *Escherichia* phage phAPEC8 |
| DW-EC-16 | 4973 | 5206 | JOODPJME_00207 hypothetical protein \| JIHLJMCN_00011 hypothetical proteinhlahoeie_00058 hypothetical protein \| DNHOGCFM_00027 hypothetical protein \| IFPLOHOB_00012 hypothetical protein \| hypothetical protein; *Escherichia* phage phAPEC8 \| hypothetical protein phAPEC8_0028; *Escherichia* phage phAPEC8 |
| DW-EC-17 | 5219 | 5692 | OAGBNOCD_00253 hypothetical protein \| Signalosome complex subunit 9; *Kluyveromyces* *lactis* (strain ATCC 8585 / CBS 2359 / DSM 70799 / NBRC 1267 / NRRL Y-1140 / WM37) |
| DW-EC-18 | 5689 | 5973 | OAGBNOCD_00254 hypothetical protein \| HFBDACEP_00013 hypothetical protein \| JIHLJMCN_00012 hypothetical protein \| BDMKCPGI_00011 hypothetical protein \| HLAHOEIE_00059 hypothetical protein; |
| DW-EC-19 | 5989 | 6165 | JIHLJMCN_00015 hypothetical protein \| HLAHOEIE_00062 hypothetical protein \| DNHOGCFM_00028 hypothetical protein \| hypothetical protein phAPEC8_0029; *Escherichia* phage phAPEC8 \| IFPLOHOB_00014 hypothetical protein \| hypothetical protein; *Escherichia* phage phAPEC8 \| Tryptophan synthase alpha chain; *Desulfovibrio vulgaris* subsp. *Vulgaris* (strain DP4) Tryptophan synthase alpha chain, *Desulfovibrio vulgaris* (strain Hildenborough / ATCC 29579 / DSM 644 / NCIMB 8303) |
| DW-EC-20 | 6166 | 6471 | OAGBNOCD_00256 hypothetical protein \| HFBDACEP_00015 hypothetical protein \| JOODPJME_00205 hypothetical protein \| JIHLJMCN_00016 hypothetical protein \| HLAHOEIE_00063 hypothetical protein \| hypothetical protein; *Escherichia* phage phAPEC8 \| hypothetical protein phAPEC8_0030; *Escherichia* phage phAPEC8 |
| DW-EC-21 | 6471 | 6671 | JOODPJME_00204 hypothetical protein \| JIHLJMCN_00017 hypothetical protein \| OAGBNOCD_00257 hypothetical protein \| HLAHOEIE_00064 hypothetical protein \| DNHOGCFM_00030 hypothetical protein \| hypothetical protein; *Escherichia* phage phAPEC8 |
| DW-EC-22 | 6686 | 6874 | HFBDACEP_00017 hypothetical protein \| JOODPJME_00203 hypothetical protein \| JIHLJMCN_00018 hypothetical protein \| IFPLOHOB_00017 hypothetical protein OAGBNOCD_00258 hypothetical protein \| hypothetical protein; *Escherichia* phage phAPEC8 |
| DW-EC-23 | 6876 | 7097 | OAGBNOCD_00259 hypothetical protein \| HFBDACEP_00018 hypothetical protein \| DNHOGCFM_00032 hypothetical protein \| hypothetical protein phAPEC8_0033; *Escherichia* phage phAPEC8 \| BDMKCPGI_00018 hypothetical protein \| hypothetical protein; *Escherichia* phage phAPEC8 \| hypothetical protein phAPEC8_0033; *Escherichia* phage phAPEC8 |
| DW-EC-24 | 7100 | 7306 | JOODPJME_00201 hypothetical protein \| BDMKCPGI_00019 hypothetical protein \| JIHLJMCN_00020 hypothetical protein \| HLAHOEIE_00067 hypothetical protein \| IFPLOHOB_00019 hypothetical protein \| hypothetical protein; *Escherichia* phage phAPEC8 |
| DW-EC-25 | 7402 | 7800 | HLAHOEIE_00068 hypothetical protein \| JOODPJME_00200 hypothetical protein \| HFBDACEP_00020 hypothetical protein \| OAGBNOCD_00261 hypothetical protein \| DNHOGCFM_00034 hypothetical protein \| hypothetical protein; *Escherichia* phage phAPEC8 |
| DW-EC-26 | 7811 | 8257 | BDMKCPGI_00021 hypothetical protein \| HLAHOEIE_00069 hypothetical protein \| HFBDACEP_00021 hypothetical protein \| JOODPJME_00199 hypothetical protein \| OAGBNOCD_00262 hypothetical protein \| hypothetical protein; *Escherichia* phage phAPEC8 \| hypothetical protein BPABA14_00370; *Acinetobacter* phage YMC-13-01-C62 \| hypothetical protein ABA1215_00290; *Acinetobacter* phage YMC11/12/R1215 |
| DW-EC-27 | 8407 | 8889 | JIHLJMCN_00024 hypothetical protein \| JOODPJME_00197 hypothetical protein \| HLAHOEIE_00071 hypothetical protein \| BDMKCPGI_00023 hypothetical protein \| HFBDACEP_00023 hypothetical protein \| hypothetical protein; *Escherichia* phage phAPEC8 |
| DW-EC-28 | 8886 | 9086 | JOODPJME_00196 hypothetical protein \| JIHLJMCN_00025 hypothetical protein \| HFBDACEP_00024 hypothetical protein \| IFPLOHOB_00024 hypothetical protein \| HLAHOEIE_00072 hypothetical protein \| hypothetical protein; *Escherichia* phage phAPEC8 |
| DW-EC-29 | 9149 | 9421 | JIHLJMCN_00026 hypothetical proteindnhogcfm_00039 hypothetical protein \| hypothetical protein phAPEC8_0040; *Escherichia* phage phAPEC8 \| HLAHOEIE_00073 hypothetical protein \| BDMKCPGI_00025 hypothetical protein \| hypothetical protein; *Escherichia* phage phAPEC8 \| hypothetical protein phAPEC8_0040; *Escherichia* phage phAPEC8 |
| DW-EC-30 | 9387 | 9713 | DNHOGCFM_00040 hypothetical protein \| hypothetical protein phAPEC8_0041; *Escherichia* phage phAPEC8 \| HFBDACEP_00026 hypothetical protein \| JIHLJMCN_00027 hypothetical protein \| HLAHOEIE_00074 hypothetical protein \| hypothetical protein; *Escherichia* phage phAPEC8 \| hypothetical protein phAPEC8_0041; *Escherichia* phage phAPEC8 |
| DW-EC-31 | 9713 | 9940 | JIHLJMCN_00028 hypothetical protein \| hypothetical protein; *Escherichia* phage phAPEC8 |
| DW-EC-32 | 9943 | 10155 | DNHOGCFM_00042 hypothetical protein \| hypothetical protein phAPEC8_0043; *Escherichia* phage phAPEC8 \| hypothetical protein; *Escherichia* phage phAPEC8 |
| DW-EC-33 | 10152 | 10472 | OAGBNOCD_00268 hypothetical protein \| JIHLJMCN_00030 hypothetical proteindnhogcfm_00043 hypothetical protein \| hypothetical protein phAPEC8_0044; *Escherichia* phage phAPEC8 \| hypothetical protein; *Escherichia* phage phAPEC8 \| hypothetical protein phAPEC8_0044; *Escherichia* phage phAPEC8 |
| DW-EC-34 | 10527 | 10940 | HLAHOEIE_00077 hypothetical protein \| OAGBNOCD_00269 hypothetical protein \| BDMKCPGI_00029 hypothetical protein |
| DW-EC-35 | 10993 | 11292 | JOODPJME_00190 hypothetical protein \| JIHLJMCN_00032 hypothetical protein \| DNHOGCFM_00045 hypothetical protein \| hypothetical protein phAPEC8_0046; *Escherichia* phage phAPEC8 \| IFPLOHOB_00031 hypothetical protein \| hypothetical protein; *Escherichia* phage phAPEC8 |
| DW-EC-36 | 11303 | 11626 | JOODPJME_00189 hypothetical protein \| JIHLJMCN_00033 hypothetical protein \| OAGBNOCD_00271 hypothetical protein \| HLAHOEIE_00079 hypothetical protein \| BDMKCPGI_00031 hypothetical protein \| hypothetical protein; *Escherichia* phage phAPEC8 |
| DW-EC-37 | 11627 | 11839 | OAGBNOCD_00272 hypothetical protein \| DNHOGCFM_00047 hypothetical protein \| hypothetical protein phAPEC8_0048; *Escherichia* phage phAPEC8 \| JOODPJME_00188 hypothetical protein \| JIHLJMCN_00034 hypothetical protein \| hypothetical protein; *Escherichia* phage phAPEC8 \| hypothetical protein; *Yersinia* phage phid1 \| Phage protein; ACLAME_Phage_proteins_with_unknown_functions Phage_cyanophage Phage_experimental; T4-like viruses *Enterobacteria* phage RB51 \| Y06C_BPT4 Uncharacterized 8.5 kda protein in tk-vs intergenic region; *Enterobacteria* phage T4 |
| DW-EC-38 | 11910 | 12581 | JOODPJME_00187 hypothetical protein \| JIHLJMCN_00035 hypothetical proteinoagbnocd_00273 hypothetical protein \| HLAHOEIE_00081 hypothetical protein \| BDMKCPGI_00033 hypothetical protein \| hypothetical protein; *Escherichia* phage phAPEC8 \| hypothetical protein phAPEC8_0049; *Escherichia* phage phAPEC8 |
| DW-EC-39 | 12590 | 13012 | OAGBNOCD_00274 hypothetical protein \| DNHOGCFM_00049 hypothetical protein \| hypothetical protein phAPEC8_0050; *Escherichia* phage phAPEC8 \| HLAHOEIE_00082 hypothetical protein \| JOODPJME_00186 hypothetical protein \| hypothetical protein; *Escherichia* phage phAPEC8 |
| DW-EC-40 | 12990 | 13355 | OAGBNOCD_00275 hypothetical protein \| HLAHOEIE_00083 hypothetical protein \| JOODPJME_00185 hypothetical proteinhfbdacep_00037 hypothetical protein \| DNHOGCFM_00050 hypothetical protein \| hypothetical protein; *Escherichia* phage phAPEC8 |
| DW-EC-41 | 13355 | 13627 | JOODPJME_00184 hypothetical protein \| JIHLJMCN_00038 hypothetical protein \| HFBDACEP_00038 hypothetical protein \| DNHOGCFM_00051 hypothetical protein \| hypothetical protein phAPEC8_0052; *Escherichia* phage phAPEC8 \| hypothetical protein; *Escherichia* phage phAPEC8 |
| DW-EC-42 | 13632 | 14243 | BDMKCPGI_00037 hypothetical protein \| HFBDACEP_00039 hypothetical protein \| IFPLOHOB_00038 hypothetical protein \| JOODPJME_00183 hypothetical protein \| DNHOGCFM_00052 hypothetical protein \| hypothetical protein; *Escherichia* phage phAPEC8 |
| DW-EC-43 | 14295 | 14633 | HLAHOEIE_00086 hypothetical protein \| DNHOGCFM_00053 hypothetical protein \| hypothetical protein phAPEC8_0054; *Escherichia* phage phAPEC8 \| JOODPJME_00182 hypothetical protein \| BDMKCPGI_00038 hypothetical protein \| hypothetical protein; *Escherichia* phage phAPEC8 , complete genome \| hypothetical protein phAPEC8_0054; *Escherichia* phage phAPEC8 |
| DW-EC-44 | 14620 | 14823 | JOODPJME_00181 hypothetical protein \| JIHLJMCN_00041 hypothetical protein \| HLAHOEIE_00087 hypothetical protein \| HFBDACEP_00041 hypothetical protein \| DNHOGCFM_00054 hypothetical protein \| hypothetical protein; *Escherichia* phage phAPEC8 |
| DW-EC-45 | 14816 | 14950 | JOODPJME_00180 hypothetical protein \| JIHLJMCN_00042 hypothetical protein \| OAGBNOCD_00280 hypothetical protein \| HLAHOEIE_00088 hypothetical protein \| BDMKCPGI_0004 hypothetical protein \| hypothetical protein; *Escherichia* phage phAPEC8 |
| DW-EC-46 | 14973 | 15392 | JOODPJME_00179 hypothetical protein \| HFBDACEP_00043 hypothetical protein \| BDMKCPGI_00041 hypothetical protein \| JIHLJMCN_00043 hypothetical protein \| IFPLOHOB_00041 hypothetical protein \| hypothetical protein; *Escherichia* phage phAPEC8 |
| DW-EC-47 | 15386 | 15595 | JIHLJMCN_00044 hypothetical protein \| HLAHOEIE_00090 hypothetical protein \| IFPLOHOB_00042 hypothetical protein \| OAGBNOCD_00282 hypothetical protein \| BDMKCPGI_00042 hypothetical protein \| hypothetical protein; *Escherichia* phage phAPEC8 |
| DW-EC-48 | 15720 | 15815 |  |
| DW-EC-49 | 15956 | 16171 | JOODPJME_00177 hypothetical protein \| JIHLJMCN_00045 hypothetical protein \| BDMKCPGI_00043 hypothetical protein \| HFBDACEP_00045 hypothetical protein \| DNHOGCFM_00058 hypothetical protein \| hypothetical protein; *Escherichia* phage phAPEC8 |
| DW-EC-50 | 16185 | 16403 | JOODPJME_00176 hypothetical protein \| JIHLJMCN_00046 hypothetical protein \| OAGBNOCD_00001 hypothetical protein \| HLAHOEIE_00092 hypothetical protein \| BDMKCPGI_00044 hypothetical protein \| hypothetical protein; *Escherichia* phage phAPEC8 \| hypothetical protein Syn1_159; Prochlorococcus phage Syn1 |
| DW-EC-51 | 16406 | 16603 | DNHOGCFM_00060 hypothetical protein \| hypothetical protein phAPEC8_0062; *Escherichia* phage phAPEC8 \| JOODPJME_00175 hypothetical protein \| JIHLJMCN_00047 hypothetical protein \| OAGBNOCD_00002 hypothetical protein \| hypothetical protein; *Escherichia* phage phAPEC8 |
| DW-EC-52 | 16587 | 17036 | DNHOGCFM_00061 hypothetical protein \| hypothetical protein phAPEC8_0063; *Escherichia* phage phAPEC8 \| JOODPJME_00174 hypothetical protein \| JIHLJMCN_00048 hypothetical protein \| BDMKCPGI_00046 hypothetical protein \| hypothetical protein; *Escherichia* phage phAPEC8 |
| DW-EC-53 | 17036 | 17200 | JOODPJME_00173 hypothetical protein \| OAGBNOCD_00004 hypothetical protein \| HLAHOEIE_00094 hypothetical protein \| BDMKCPGI_00047 hypothetical protein \| HFBDACEP_00049 hypothetical protein \| hypothetical protein; *Escherichia* phage phAPEC8 \| hypothetical protein phAPEC8_0064; *Escherichia* phage phAPEC8 |
| DW-EC-54 | 17197 | 17460 | OAGBNOCD_00005 hypothetical protein \| HLAHOEIE_00095 hypothetical protein \| DNHOGCFM_00063 hypothetical protein \| hypothetical protein phAPEC8_0065; *Escherichia* phage phAPEC8 \| JIHLJMCN_00050 hypothetical protein \| hypothetical protein; *Escherichia* phage phAPEC8 |
| DW-EC-55 | 17469 | 18077 | JOODPJME_00171 hypothetical protein \| JIHLJMCN_00051 hypothetical protein \| BDMKCPGI_00049 hypothetical protein \| HFBDACEP_00051 hypothetical protein \| OAGBNOCD_00006 hypothetical protein \| hypothetical protein; *Escherichia* phage phAPEC8 |
| DW-EC-56 | 18087 | 18188 | JOODPJME_00170 hypothetical protein \| JIHLJMCN_00052 hypothetical protein \| OAGBNOCD_00007 hypothetical protein \| HLAHOEIE_00097 hypothetical protein \| BDMKCPGI_00050 hypothetical protein \| hypothetical protein; *Escherichia* phage phAPEC8 \| ZN778_HUMAN Zinc finger protein 77; *Homo sapiens* |
| DW-EC-57 | 18241 | 18414 | JOODPJME_00169 hypothetical protein \| JIHLJMCN_00053 hypothetical protein \| OAGBNOCD_00008 hypothetical protein \| HLAHOEIE_00098 hypothetical protein \| BDMKCPGI_00051 hypothetical protein \| hypothetical protein; *Escherichia* phage phAPEC8 \| hypothetical protein phAPEC8_0068; *Escherichia* phage phAPEC8 |
| DW-EC-58 | 18427 | 18708 | JOODPJME_00168 hypothetical protein \| JIHLJMCN_00054 hypothetical protein \| BDMKCPGI_00052 hypothetical protein \| HFBDACEP_00054 hypothetical protein \| DNHOGCFM_00067 hypothetical protein \| hypothetical protein; *Escherichia* phage phAPEC8 \| major capsid protein; *Bacillus* phage VMY22 |
| DW-EC-59 | 18800 | 19030 | JOODPJME_00167 hypothetical protein |
| DW-EC-60 | 19115 | 19372 | JIHLJMCN_00056 hypothetical protein \| OAGBNOCD_00011 hypothetical protein \| HLAHOEIE_00101 hypothetical protein \| HFBDACEP_00056 hypothetical protein \| DNHOGCFM_00069 hypothetical protein \| hypothetical protein; *Escherichia* phage phAPEC8 |
| DW-EC-61 | 19416 | 20231 | JOODPJME_00165; NAD-dependent protein deacylase \| IFPLOHOB_00055; NAD-dependent protein deacylase \| JIHLJMCN_00057; NAD-dependent protein deacylase \| HLAHOEIE_00102; NAD-dependent protein  deacylase \| HFBDACEP_00057; NAD-dependent  protein deacylase \| NAD-dependent protein deacetylase of SIR2 family; *Enterobacteria* phage phi92 \| putative Sir2-like transferase; *Enterobacteria*  phage ECGD1 \| Phi92_gp038; *Enterobacteria* phage phi92 |
| DW-EC-62 | 20216 | 20701 | JOODPJME_00164 hypothetical protein \| JIHLJMCN_00058 hypothetical proteinjihljmcn_00057; NAD-dependent protein deacylaseifplohob_00056 hypothetical protein \| HLAHOEIE_00103 hypothetical protein \| hypothetical protein; *Escherichia* phage phAPEC8 |
| DW-EC-63 | 20760 | 21413 | HLAHOEIE_00104 hypothetical protein \| DNHOGCFM_00072 hypothetical protein \| hypothetical protein phAPEC8_0074; *Escherichia* phage phAPEC8 \| JIHLJMCN_00059 hypothetical protein \| OAGBNOCD_00014 hypothetical protein \| hypothetical protein; *Escherichia* phage phAPEC8 \| hypothetical protein phAPEC8_0074 ; *Escherichia* phage phAPEC8 |
| DW-EC-64 | 21413 | 22096 | JOODPJME_00162 hypothetical proteinjihljmcn_00060 hypothetical proteinoagbnocd_00015 hypothetical protein \| HLAHOEIE_00105 hypothetical protein \| BDMKCPGI_00058 hypothetical protein \| hypothetical protein; *Escherichia* phage phAPEC8 \| hypothetical protein phAPEC8_0075; *Escherichia* phage phAPEC8 |
| DW-EC-65 | 22089 | 22484 | JOODPJME_00161 hypothetical protein \| JIHLJMCN_00061 hypothetical protein \| OAGBNOCD_00016 hypothetical protein \| HLAHOEIE_00106 hypothetical protein \| BDMKCPGI_00059 hypothetical protein \| hypothetical protein; *Escherichia* phage phAPEC8 \| ORF005; *Staphylococcus* phage 2638A |
| DW-EC-66 | 22486 | 23427 | OAGBNOCD_00017 hypothetical protein \| DNHOGCFM_00075 hypothetical protein \| hypothetical protein phAPEC8_0077; *Escherichia* phage phAPEC8 \| JOODPJME_00160 hypothetical protein \| HLAHOEIE_00107 hypothetical protein \| hypothetical protein; *Escherichia* phage phAPEC8 |
| DW-EC-67 | 23518 | 23790 | JOODPJME_00159 hypothetical protein \| JIHLJMCN_00063 hypothetical protein \| OAGBNOCD_00018 hypothetical proteinhlahoeie_00108 hypothetical protein \| BDMKCPGI_00061 hypothetical protein \| hypothetical protein; *Escherichia* phage phAPEC8 \| hypothetical protein phAPEC8_0078; *Escherichia* phage phAPEC8 \| alpha-mannosidase (EC 3.2.1.24); mannosyl-oligosaccharide alpha-1,2-mannosidase (EC 3.2.1.113); mannosyl-oligosaccharide alpha-1,3-1,6-mannosidase (EC  3.2.1.114); alpha-2-O-mannosylglycerate hydrolase (EC 3.2.1.170); mannosyl-oligosaccharide alpha-1,3-mannosidase (EC 3.2.1.-) |
| DW-EC-68 | 23800 | 24018 | JOODPJME_00158 hypothetical proteinjihljmcn_00064 hypothetical protein \| OAGBNOCD_00019 hypothetical protein \| IFPLOHOB_00062 hypothetical protein \| DNHOGCFM_00077 hypothetical protein \| hypothetical protein; *Escherichia* phage phAPEC8 \| hypothetical protein phAPEC8_0079; *Escherichia* phage phAPEC8 |
| DW-EC-69 | 24018 | 24293 | IFPLOHOB_00063 hypothetical protein \| JOODPJME_00157 hypothetical protein \| JIHLJMCN_00065 hypothetical protein \| Phage protein;  ACLAME_Phage_proteins_with_unknown_functions Phage_cyanophage  Phage_experimental; *Enterobacteria* phage CC31 \| Phage protein; ACLAME_Phage_proteins_with_unknown_functions Phage_cyanophage  Phage_experimental; *Enterobacteria* phage IME08 \| Phage protein ; ACLAME_Phage_proteins_with_unknown_functions Phage_cyanophage  Phage_experimental; T4-like viruses *Enterobacteria* phage  JS98 \| Phage protein;  ACLAME_Phage_proteins_with_unknown_functions Phage_cyanophage  Phage_experimental; T4-like viruses *Enterobacteria* phage  JS10 \| hypothetical  protein; *Enterobacteria* phage Bp7 \| Uncharacterized 10.2 kda protein in regb-denv intergenic region  ; *Enterobacteria* phage t4hypothetical  protein VR20_114; *Escherichia* phage vb_ecom_VR20 \| hypothetical  protein CC31p124; Enterobacter phage CC31 \| Vs.4 gene product; *Enterobacteria* phage  IME08 \| Vs.4 conserved  hypothetical protein; *Enterobacteria* phage JS10 \| Vs.4 conserved  hypothetical protein; *Escherichia* phage JS98 |
| DW-EC-70 | 24305 | 24502 | JOODPJME_00156 hypothetical protein \| JIHLJMCN_00066 hypothetical protein \| HLAHOEIE_00111 hypothetical protein \| BDMKCPGI_00064 hypothetical protein \| IFPLOHOB_00064 hypothetical protein \| hypothetical protein; *Escherichia* phage phAPEC8 \| hypothetical protein phAPEC8_0081; *Escherichia* phage phAPEC8 |
| DW-EC-71 | 24486 | 24776 | JOODPJME_00155 hypothetical protein \| JIHLJMCN_00067 hypothetical protein \| OAGBNOCD_00022 hypothetical protein \| HLAHOEIE_00112 hypothetical protein \| HFBDACEP_00067 hypothetical protein \| hypothetical protein; *Escherichia* phage phAPEC8 \| hypothetical protein phAPEC8_0082; *Escherichia* phage phAPEC8 |
| DW-EC-72 | 24755 | 25204 | HLAHOEIE_00113 hypothetical protein \| JOODPJME_00154 hypothetical protein \| JIHLJMCN_00068 hypothetical proteinbdmkcpgi_00066 hypothetical protein \| HFBDACEP_00068 hypothetical protein \| hypothetical protein; *Escherichia* phage phAPEC8 |
| DW-EC-73 | 25257 | 25838 | JOODPJME_00153 hypothetical protein \| JIHLJMCN_00069 hypothetical protein \| OAGBNOCD_00024 hypothetical protein \| HFBDACEP_00069 hypothetical protein \| DNHOGCFM_00082 hypothetical protein \| hypothetical protein; *Escherichia* phage phAPEC8 \| hypothetical protein phAPEC8_0084; *Escherichia* phage phAPEC8 |
| DW-EC-74 | 25846 | 26154 | JOODPJME_00152 hypothetical protein \| JIHLJMCN_00070 hypothetical protein \| OAGBNOCD_00025 hypothetical protein \| HLAHOEIE_00115 hypothetical protein \| BDMKCPGI_00068 hypothetical protein \| hypothetical protein; *Escherichia* phage phAPEC8 |
| DW-EC-75 | 26154 | 26897 | JOODPJME_00151 Serine/threonine-protein phosphatase 2 \| JIHLJMCN_00071 Serine/threonine-protein phosphatase 2 \| OAGBNOCD_00026 Serine/threonine-protein phosphatase 2 \| BDMKCPGI_00069 Serine/threonine-protein phosphatase 2 \| HFBDACEP_00071 Serine/threonine-protein phosphatase 2 \| putative serine/threonine protein phosphatase; *Escherichia* phage  phAPEC8 , complete genome |
| DW-EC-76 | 26894 | 27223 | JIHLJMCN_00072 hypothetical protein \| DNHOGCFM_00085 hypothetical protein \| hypothetical protein phAPEC8_0087; *Escherichia* phage phAPEC8 \| JOODPJME_00150 hypothetical protein \| OAGBNOCD_00027 hypothetical protein \| hypothetical protein; *Escherichia* phage phAPEC8 |
| DW-EC-77 | 27234 | 27806 | DNHOGCFM_00086 hypothetical protein \| hypothetical protein phAPEC8_0088; *Escherichia* phage phAPEC8 \| JOODPJME_00149 hypothetical protein \| JIHLJMCN_00073 hypothetical protein \| HLAHOEIE_00118 hypothetical protein \| hypothetical protein; *Escherichia* phage phAPEC8 |
| DW-EC-78 | 27821 | 28015 | JOODPJME_00148 hypothetical protein \| JIHLJMCN_00074 hypothetical protein \| OAGBNOCD_00029 hypothetical protein \| HLAHOEIE_00119 hypothetical protein \| BDMKCPGI_00072 hypothetical protein \| hypothetical protein; *Escherichia* phage phAPEC8 \| hypothetical protein JWAP_00025; *Achromobacter* phage 83-24 |
| DW-EC-79 | 28061 | 28264 | JOODPJME_00147 hypothetical protein \| JIHLJMCN_00075 hypothetical protein \| HLAHOEIE_00120 hypothetical protein \| BDMKCPGI_00073 hypothetical protein \| HFBDACEP_00075 hypothetical protein \| hypothetical protein; *Escherichia* phage phAPEC8 |
| DW-EC-80 | 28264 | 28500 | JOODPJME_00146 hypothetical protein \| JIHLJMCN_00076 hypothetical protein \| HLAHOEIE_00121 hypothetical protein \| BDMKCPGI_00074 hypothetical protein \| HFBDACEP_00076 hypothetical protein \| hypothetical protein; *Escherichia* phage phAPEC8 |
| DW-EC-81 | 28513 | 29022 | JOODPJME_00145 hypothetical protein \| JIHLJMCN_00077 hypothetical protein \| OAGBNOCD_00032 hypothetical protein \| HLAHOEIE_00122 hypothetical protein \| BDMKCPGI_00075 hypothetical protein \| hypothetical protein; *Escherichia* phage phAPEC8 \| hypothetical protein phAPEC8_0092; *Escherichia* phage phAPEC8 |
| DW-EC-82 | 29006 | 29440 | JOODPJME_00144 hypothetical protein \| JIHLJMCN_00078 hypothetical protein \| HLAHOEIE_00123 hypothetical protein \| BDMKCPGI_00076 hypothetical protein \| HFBDACEP_00078 hypothetical protein \| hypothetical protein; *Escherichia* phage phAPEC8 |
| DW-EC-83 | 29486 | 30178 | JOODPJME_00143 ATP-dependent protease subunit hslv \| JIHLJMCN_00079 ATP-dependent protease subunit hslv \| OAGBNOCD_00034 ATP-dependent protease subunit hslv \| HLAHOEIE_00124 ATP-dependent  protease subunit \| HSLVBDMKCPGI_00077 ATP-dependent protease subunit hslv \| hypothetical protein; *Escherichia* phage phAPEC8 \| hypothetical  protein phAPEC8_0094; *Escherichia* phage phAPEC8 |
| DW-EC-84 | 30178 | 30678 | JOODPJME_00142 hypothetical protein \| JIHLJMCN_00080 hypothetical protein \| OAGBNOCD_00035 hypothetical protein \| HLAHOEIE_00125 hypothetical protein \| BDMKCPGI_00078 hypothetical protein \| hypothetical protein; *Escherichia* phage phAPEC8 |
| DW-EC-85 | 30714 | 31472 | JOODPJME_00141 PhoH-like protein \| JIHLJMCN_00081 PhoH -like protein \| OAGBNOCD_00036 PhoH -like protein \| HLAHOEIE_00126 PhoH -like protein \| BDMKCPGI_00079 PhoH -like protein \| putative PhoH family  protein; *Escherichia* phage phAPEC8 \| putative PhoH family protein; *Escherichia*  phage phAPEC8 |
| DW-EC-86 | 31510 | 32010 | JIHLJMCN_00082 hypothetical protein \| HLAHOEIE_00127 hypothetical protein \| BDMKCPGI_00080 hypothetical protein \| HFBDACEP_00082 hypothetical protein \| IFPLOHOB_00080 hypothetical proteinputative bacteriophage  T4-like lysozyme; *Escherichia* phage  phAPEC8 \| putative bacteriophage T4-like lysozymelysozyme (EC 3.2.1.17) \| lysozyme (EC 3.2.1.17) \| lysozyme (EC 3.2.1.17) \| lysozyme (EC 3.2.1.17) \| lysozyme (EC 3.2.1.17) |
| DW-EC-87 | 32018 | 32167 | JOODPJME_00139; hypothetical protein \| JIHLJMCN_00083 hypothetical protein \| OAGBNOCD_00038 hypothetical protein \| HLAHOEIE_00128 hypothetical protein \| BDMKCPGI_00081 hypothetical protein \| hypothetical protein; *Escherichia* phage phAPEC8 |
| DW-EC-88 | 32173 | 32433 | JOODPJME_00138 hypothetical protein \| JIHLJMCN_00084 hypothetical protein \| OAGBNOCD_00039 hypothetical protein \| HLAHOEIE_00129 hypothetical protein \| HFBDACEP_00084 hypothetical protein \| putative glutaredoxin; *Escherichia* phage phAPEC8 \| putative glutaredoxin 1; *Escherichia* phage phAPEC8 |
| DW-EC-89 | 32433 | 33275 | HLAHOEIE_00130 dTDP-4-dehydrorhamnose reductase \| BDMKCPGI_00083 dTDP-4-dehydrorhamnose reductase \| JOODPJME_00137 dTDP-4-dehydrorhamnose reductase \| OAGBNOCD_00040  dTDP-4-dehydrorhamnose reductase \| DNHOGCFM_00098 dTDP-4-dehydrorhamnose reductase \| putative dTDP-4-dehydrorhamnose reductase; *Escherichia* phage phAPEC8 |
| DW-EC-90 | 33269 | 33835 | JOODPJME_00136 dTDP-4-dehydrorhamnose 3,5-epimerase \| JIHLJMCN_00086 dTDP-4-dehydrorhamnose 3,5-epimerase \| IFPLOHOB_00084 dTDP-4-dehydrorhamnose 3,5-epimerase \| DNHOGCFM_00099  dTDP-4-dehydrorhamnose 3,5-epimerase \| putative dTDP-4-dehydrorhamnose 3,5-epimerase; *Escherichia* phage phAPEC8 \| putative dTDP-4-dehydrorhamnose 3,5-epimerase; *Escherichia* phage phAPEC8 \| putative dTDP-4-dehydrorhamnose 3,5-epimerase; *Escherichia* phage  phAPEC8 |
| DW-EC-91 | 33845 | 34171 | OAGBNOCD_00042 hypothetical protein \| DCKEIKPP_00194 hypothetical protein \| JIHLJMCN_00089 hypothetical protein \| DNHOGCFM_00102 hypothetical protein \| IFPLOHOB_00087 hypothetical protein \| hypothetical protein; *Escherichia* phage phAPEC8 \| Phage  protein; ACLAME_Phage_proteins_with_unknown_functions Phage_cyanophage Phage_experimental; *Enterobacteria* phage phi92 |
| DW-EC-92 | 34161 | 34322 | JIHLJMCN_00090 hypothetical protein \| HLAHOEIE_00135 hypothetical protein \| HFBDACEP_00090 hypothetical protein \| IFPLOHOB_00088 hypothetical proteinoagbnocd_00043 hypothetical protein \| hypothetical protein; *Escherichia* phage phAPEC8 |
| DW-EC-93 | 34312 | 34464 | JOODPJME_00131 hypothetical protein \| JIHLJMCN_00091 hypothetical protein \| HLAHOEIE_00136 hypothetical protein \| BDMKCPGI_00089 hypothetical protein \| HFBDACEP_00091 hypothetical protein \| hypothetical protein; *Escherichia* phage phAPEC8 \| Chaperone  protein DNAJ; *ThioBacillus denitrificans* (strain ATCC 25259) |
| DW-EC-94 | 34464 | 34673 | JOODPJME_00130 hypothetical protein \| OAGBNOCD_00045 hypothetical protein \| DNHOGCFM_00105 hypothetical protein \| hypothetical protein phAPEC8_00107; *Escherichia* phage phAPEC8 \| JIHLJMCN_00092 hypothetical protein \| hypothetical protein; *Escherichia* phage phAPEC8 \| hypothetical protein phAPEC8_00107; *Escherichia* phage phAPEC8 |
| DW-EC-95 | 34673 | 35758 | JIHLJMCN_00093 Ribonucleoside-diphosphate reductase 1 subunit beta \| HLAHOEIE_00138 Ribonucleoside-diphosphate reductase 1 subunit beta \| BDMKCPGI_00091 Ribonucleoside-diphosphate  reductase 1 subunit beta \| HFBDACEP_00093 Ribonucleoside-diphosphate reductase 1 subunit beta \| IFPLOHOB_00091 Ribonucleoside-diphosphate reductase 1 subunit betaputative ribonucleotide reductase of class Ia (aerobic) beta subunit; *Escherichia* phage phAPEC8 \| putative ribonucleotide reductase of class Ia (aerobic) beta subunit; *Escherichia* phage phAPEC8 |
| DW-EC-96 | 35745 | 36083 | Hypothetical protein phAPEC8_00109; *Escherichia* phage phAPEC8 \| DNHOGCFM_00107 hypothetical protein \| OAGBNOCD_00047 hypothetical protein \| BDMKCPGI_00092 hypothetical protein \| hypothetical protein; *Escherichia* phage phAPEC8 |
| DW-EC-97 | 36124 | 38364 | JIHLJMCN_00094 Ribonucleoside-diphosphate reductase 1 subunit alpha \| IFPLOHOB_00092 Ribonucleoside-diphosphate reductase 1 subunit alpha \| DNHOGCFM_00108 Ribonucleoside-diphosphate  reductase 1 subunit alpha \| putative ribonucleotide reductase of class Ia (aerobic) alpha subunit; *Escherichia* phage phAPEC8 \| HLAHOEIE_00139 Ribonucleoside-diphosphate  reductase 1 subunit alpha \| putative ribonucleotide reductase of class Ia  (aerobic) alpha subunit; *Escherichia* phage phAPEC8 \| putative ribonucleotide reductase of class Ia (aerobic) alpha subunit; *Escherichia* phage phAPEC8 |
| DW-EC-98 | 38381 | 38635 | JOODPJME_00127 hypothetical protein \| JIHLJMCN_00095 hypothetical protein \| OAGBNOCD_00049 hypothetical protein \| HLAHOEIE_00140 hypothetical proteinhfbdacep_00096 hypothetical protein \| hypothetical protein; *Escherichia* phage phAPEC8 \| Phage protein; ACLAME_Phage_proteins_with_unknown_functions Phage_cyanophage Phage_experimental; *Enterobacteria* phage phi92 |
| DW-EC-99 | 38632 | 39042 | JOODPJME_00126 hypothetical protein \| JIHLJMCN_00096 hypothetical protein \| HLAHOEIE_00141 hypothetical protein \| BDMKCPGI_00096 hypothetical protein \| HFBDACEP_00097 hypothetical protein \| hypothetical protein; *Escherichia* phage phAPEC8 |
| DW-EC-100 | 39141 | 40139 | BDMKCPGI_00097 hypothetical protein \| JIHLJMCN_00097 hypothetical protein \| HLAHOEIE_00142 hypothetical protein \| IFPLOHOB_00095 hypothetical protein \| OAGBNOCD_00051 hypothetical protein \| putative thymidylate synthase; *Escherichia* phage phAPEC8 \| putative thymidylate synthase; *Escherichia* phage phAPEC8 |
| DW-EC-101 | 40149 | 40757 | JOODPJME_00124 hypothetical protein \| JIHLJMCN_00098 hypothetical protein \| HLAHOEIE_00143 hypothetical protein \| BDMKCPGI_00098 hypothetical protein \| HFBDACEP_00099 hypothetical protein \| hypothetical protein; *Escherichia* phage phAPEC8 \| hypothetical protein phAPEC8_00114; *Escherichia* phage phAPEC8 |
| DW-EC-102 | 40794 | 41348 | OAGBNOCD_00053 hypothetical protein \| DNHOGCFM_00113 hypothetical protein \| hypothetical protein phAPEC8_00115; *Escherichia* phage phAPEC8 \| JOODPJME_00123 hypothetical protein \| JIHLJMCN_00099 hypothetical protein \| hypothetical protein; *Escherichia* phage phAPEC8 \| hypothetical protein phAPEC8_00115; *Escherichia* phage phAPEC8 |
| DW-EC-103 | 41359 | 41553 | JOODPJME_00122 hypothetical protein \| JIHLJMCN_00100 hypothetical protein \| OAGBNOCD_00054 hypothetical protein \| HLAHOEIE_00145 hypothetical protein \| BDMKCPGI_00100 hypothetical protein \| hypothetical protein; *Escherichia* phage phAPEC8 \| hypothetical protein phAPEC8_00116; *Escherichia* phage phAPEC8 |
| DW-EC-104 | 41537 | 41938 | DNHOGCFM_00115 hypothetical protein \| hypothetical protein phAPEC8_00117; *Escherichia* phage phAPEC8 \| OAGBNOCD_00055 hypothetical protein \| BDMKCPGI_00101 hypothetical protein \| HFBDACEP_00102 hypothetical protein \| hypothetical protein; *Escherichia* phage phAPEC8 |
| DW-EC-105 | 41931 | 42158 | OAGBNOCD_00056 hypothetical protein \| JIHLJMCN_00102 hypothetical protein \| HLAHOEIE_00147 hypothetical protein \| HFBDACEP_00103 hypothetical protein \| IFPLOHOB_00100 hypothetical protein \| hypothetical protein; *Escherichia* phage phAPEC8 |
| DW-EC-106 | 42245 | 43204 | JIHLJMCN_00103 hypothetical protein \| HLAHOEIE_00148 hypothetical protein \| HFBDACEP_00104 hypothetical protein \| IFPLOHOB_00101 hypothetical protein \| JOODPJME_00119 hypothetical protein \| hypothetical protein ; *Escherichia* phage phAPEC8 \| hypothetical protein phAPEC8_00119; *Escherichia* phage phAPEC8 |
| DW-EC-107 | 43201 | 43890 | JOODPJME_00118 hypothetical protein \| JIHLJMCN_00104 hypothetical protein \| OAGBNOCD_00058 hypothetical protein \| HLAHOEIE_00149 hypothetical protein \| BDMKCPGI_00104 hypothetical protein \| putative exonuclease; *Escherichia* phage phAPEC8 \| putative exonuclease; *Escherichia* phage phAPEC8 |
| DW-EC-108 | 43968 | 44090 | Hypothetical protein phAPEC8_00121; *Escherichia* phage phAPEC8 \| hypothetical protein; *Escherichia* phage phAPEC8 |
| DW-EC-109 | 44100 | 44192 |  |
| DW-EC-110 | 44332 | 44541 | JOODPJME_00117 hypothetical protein \| JIHLJMCN_00105 hypothetical protein \| HLAHOEIE_00150 hypothetical protein \| HFBDACEP_00106 hypothetical protein \| DNHOGCFM_00119 hypothetical protein \| hypothetical protein; *Escherichia* phage phAPEC8 \| hypothetical protein phAPEC8_00122; *Escherichia* phage phAPEC8 |
| DW-EC-111 | 44614 | 45492 | JOODPJME_00116 Recombination-associated protein RdgC \| JIHLJMCN_00106 Recombination-associated protein RdgC \| HLAHOEIE_00151 Recombination-associated protein RdgC \| HFBDACEP_00107 Recombination-associated protein RdgC \| IFPLOHOB_00104 Recombination-associated protein RdgC \| putative exonuclease RdgC; *Escherichia* phage phAPEC8 \| putative exonuclease RdgC; *Escherichia* phage phAPEC8 |
| DW-EC-112 | 45549 | 46274 | JOODPJME_00115 hypothetical protein \| JIHLJMCN_00107 hypothetical protein \| HLAHOEIE_00152 hypothetical protein \| HFBDACEP_00108 hypothetical protein \| DNHOGCFM_00121 hypothetical protein \| hypothetical protein; *Escherichia* phage phAPEC8 \| hypothetical protein phAPEC8_00124; *Escherichia* phage phAPEC8 |
| DW-EC-113 | 46284 | 46466 | JOODPJME_00114 hypothetical protein \| HLAHOEIE_00153 hypothetical protein \| BDMKCPGI_00108 hypothetical protein \| HFBDACEP_00109 hypothetical protein \| IFPLOHOB_00106 hypothetical protein \| hypothetical protein; *Escherichia* phage phAPEC8 |
| DW-EC-114 | 46463 | 46657 | JOODPJME_00113 hypothetical protein \| JIHLJMCN_00109 hypothetical protein \| HLAHOEIE_00154 hypothetical protein \| HFBDACEP_00110 hypothetical protein \| IFPLOHOB_00107 hypothetical protein |
| DW-EC-115 | 46667 | 47782 | DNHOGCFM_00123 hypothetical protein \| putative ATP-dependent DNA ligase; *Escherichia* phage phAPEC8 \| JIHLJMCN_00110 hypothetical protein \| OAGBNOCD_00063 hypothetical protein \| HLAHOEIE_00155 hypothetical protein \| putative ATP-dependent DNA ligase; *Escherichia* phage  phAPEC8 \| putative ATP-dependent DNA ligase; *Escherichia* phage phAPEC8 |
| DW-EC-116 | 47782 | 48003 | JOODPJME_00111 hypothetical protein \| JIHLJMCN_00111 hypothetical protein \| OAGBNOCD_00064 hypothetical protein \| HLAHOEIE_00156 hypothetical protein \| BDMKCPGI_00111 hypothetical protein \| hypothetical protein; *Escherichia* phage phAPEC8 |
| DW-EC-117 | 48000 | 48191 | OAGBNOCD_00065 hypothetical protein \| JOODPJME_00110 hypothetical protein \| JIHLJMCN_00112 hypothetical protein \| BDMKCPGI_00112 hypothetical protein \| HFBDACEP_00113 hypothetical protein |
| DW-EC-118 | 48201 | 48527 | OAGBNOCD_00066 hypothetical protein \| JOODPJME_00109 hypothetical protein \| JIHLJMCN_00113 hypothetical protein \| HLAHOEIE_00158 hypothetical protein \| BDMKCPGI_00113 hypothetical protein |
| DW-EC-119 | 48537 | 48941 | OAGBNOCD_00067 hypothetical proteinjoodpjme_00108 hypothetical proteinjihljmcn_00114 hypothetical protein \| BDMKCPGI_00114 hypothetical protein \| HFBDACEP_00115 hypothetical protein \| Phage protein; ACLAME_Phage_proteins_with_unknown_functions Phage_cyanophage  Phage_experimental; *Enterobacteria* phage phi92 \| hypothetical protein; *Cronobacter* phage vb_csam_GAP31,  complete genome \| AAA domain-containing protein; *Enterobacteria* phage 4MG \| hypothetical protein ECGD1_105 |
| DW-EC-120 | 48942 | 49874 | JOODPJME_00107 hypothetical protein \| JIHLJMCN_00115 hypothetical protein \| HLAHOEIE_00160 hypothetical protein \| IFPLOHOB_00113 hypothetical protein \| BDMKCPGI_00115 hypothetical protein \| Phage protein; ACLAME_Phage_proteins_with_unknown_functions Phage_cyanophage Phage_experimental; *Enterobacteria* phage phi92 \| hypothetical protein; *Salmonella* phage SSE-121, complete  genome \| Phage protein; ACLAME_Phage_proteins_with_unknown_functions Phage_cyanophage Phage_experimental; *Salmonella* phage PVP-SE1 \| putative RNA ligase/RNA repair; *Enterobacteria* phage ECGD1 \| Phi92_gp096; *Enterobacteria* phage phi92 \| hypothetical protein; *Salmonella* phage SSE121 \| 106 gene product; *Salmonella* phage PVP-SE1 |
| DW-EC-121 | 49876 | 50427 | OAGBNOCD_00069 hypothetical protein \| JOODPJME_00106 hypothetical protein \| JIHLJMCN_00116 hypothetical protein \| HLAHOEIE_00161 hypothetical protein \| BDMKCPGI_00116 hypothetical protein \| putative phosphoesterase or phosphohydrolase; *Enterobacteria*  phage phi92 \| hypothetical protein; *Escherichia*  phage phAPEC8 \| putative phosphoesterase; *Enterobacteria* phage  ECGD1 \| Phi92_gp097; *Enterobacteria* phage phi92 |
| DW-EC-122 | 50427 | 50783 | JOODPJME_00105 hypothetical protein \| JIHLJMCN_00117 hypothetical protein \| HLAHOEIE_00162 hypothetical protein \| BDMKCPGI_00117 hypothetical protein \| HFBDACEP_00118 hypothetical protein \| hypothetical protein; *Escherichia* phage phAPEC8 \| Phage  protein; ACLAME_Phage_proteins_with_unknown_functions Phage_cyanophage Phage_experimental; *Enterobacteria* phage phi92 \| hypothetical protein phAPEC8_00128; *Escherichia* phage phAPEC8 \| hypothetical protein ECGD1_108; *Enterobacteria* phage ECGD1 \| Phi92_gp098; *Enterobacteria* phage phi92 |
| DW-EC-123 | 50790 | 50933 | JOODPJME_00104 hypothetical protein \| HLAHOEIE_00163 hypothetical protein \| BDMKCPGI_00118 hypothetical protein \| HFBDACEP_00119 hypothetical protein \| IFPLOHOB_00116 hypothetical protein \| hypothetical protein; *Escherichia* phage phAPEC8 |
| DW-EC-124 | 50943 | 51299 | OAGBNOCD_00071 hypothetical protein \| hypothetical protein; *Escherichia* phage phAPEC8 \| hypothetical protein phAPEC8_00130; *Escherichia* phage phAPEC8 |
| DW-EC-125 | 51310 | 51780 | JOODPJME_00102 hypothetical protein \| JIHLJMCN_00120 hypothetical protein \| BDMKCPGI_00120 hypothetical protein \| HFBDACEP_00121 hypothetical protein \| IFPLOHOB_00118 hypothetical protein \| hypothetical protein; *Escherichia* phage phAPEC8 \| hypothetical protein phAPEC8_00131; *Escherichia* phage phAPEC8 |
| DW-EC-126 | 51777 | 51983 | HLAHOEIE_00166 hypothetical protein \| JOODPJME_00101 hypothetical protein \| JIHLJMCN_00121 hypothetical protein \| BDMKCPGI_00121 hypothetical protein \| DNHOGCFM_00129 hypothetical protein \| hypothetical protein; *Escherichia* phage phAPEC8 |
| DW-EC-127 | 51980 | 52162 | HLAHOEIE_00167 hypothetical proteindnhogcfm_00130 hypothetical protein \| hypothetical protein phAPEC8_00133; *Escherichia* phage phAPEC8 \| BDMKCPGI_00122 hypothetical protein \| IFPLOHOB_00120 hypothetical protein \| hypothetical protein; *Escherichia* phage phAPEC8 |
| DW-EC-128 | 52159 | 52440 | OAGBNOCD_00075 hypothetical protein \| HLAHOEIE_00168 hypothetical protein \| BDMKCPGI_00123 hypothetical protein \| DNHOGCFM_00131 hypothetical protein \| hypothetical protein phAPEC8_00134; *Escherichia* phage phAPEC8 \| hypothetical protein; *Escherichia* phage phAPEC8 \| hypothetical protein ECBP2_0039; *Escherichia* phage ECBP2 |
| DW-EC-129 | 52437 | 52601 | IFPLOHOB_00122 hypothetical protein \| JOODPJME_00098 hypothetical protein \| JIHLJMCN_00124 hypothetical protein \| HLAHOEIE_00169 hypothetical protein \| HFBDACEP_00125 hypothetical protein \| putative transposase-like protein; *Escherichia*  phage phAPEC8 \| putative transposase-like protein; *Escherichia* phage phAPEC8 |
| DW-EC-130 | 52744 | 52983 | JOODPJME_00097 hypothetical protein \| JIHLJMCN_00125 hypothetical protein \| OAGBNOCD_00077 hypothetical protein \| HLAHOEIE_00170 hypothetical protein \| BDMKCPGI_00126 hypothetical protein \| hypothetical protein; *Escherichia* phage phAPEC8 |
| DW-EC-131 | 52980 | 53174 | JOODPJME_00096 hypothetical protein \| JIHLJMCN_00126 hypothetical protein \| OAGBNOCD_00078 hypothetical protein \| HLAHOEIE_00171 hypothetical protein \| BDMKCPGI_00127 hypothetical protein \| hypothetical protein; *Escherichia* phage phAPEC8 |
| DW-EC-132 | 53171 | 53287 | JOODPJME_00095 hypothetical protein \| JIHLJMCN_00127 hypothetical protein \| OAGBNOCD_00079 hypothetical protein \| HLAHOEIE_00172 hypothetical protein \| HFBDACEP_00128 hypothetical protein \| hypothetical protein; *Escherichia* phage phAPEC8 |
| DW-EC-133 | 53271 | 53594 | OAGBNOCD_00080 hypothetical protein \| BDMKCPGI_00128 hypothetical protein \| IFPLOHOB_00126 hypothetical protein \| DNHOGCFM_00136 hypothetical protein \| hypothetical protein phAPEC8_00139; *Escherichia* phage phAPEC8 \| hypothetical protein; *Escherichia* phage phAPEC8 |
| DW-EC-134 | 53963 | 54094 |  |
| DW-EC-135 | 54065 | 54250 | JOODPJME_00093 hypothetical protein \| JIHLJMCN_00129 hypothetical protein \| OAGBNOCD_00081 hypothetical protein \| HLAHOEIE_00174 hypothetical protein \| HFBDACEP_00130 hypothetical protein \| hypothetical protein; *Escherichia* phage phAPEC8 \| hypothetical protein; *Cronobacter*  phage vb_csam_GAP31 \| hypothetical protein GAP31_183; *Cronobacter*  phage vb_csam_GAP31 |
| DW-EC-136 | 54288 | 54539 | Hypothetical protein phAPEC8_00141; *Escherichia* phage phAPEC8 \| hypothetical protein; *Escherichia* phage phAPEC8 |
| DW-EC-137 | 54548 | 54796 | JOODPJME_00092 hypothetical protein \| OAGBNOCD_00082 hypothetical protein \| HLAHOEIE_00175 hypothetical protein \| BDMKCPGI_00130 hypothetical protein \| HFBDACEP_00131 hypothetical protein \| hypothetical protein; *Escherichia* phage phAPEC8 \| T7-like phage ssDNA-binding protein; ACLAME_Phage_replication; *Pseudomonas* phage phi15 \| putative ssDNA -binding protein; *Pseudomonas*  phage phi15 |
| DW-EC-138 | 54869 | 54973 |  |
| DW-EC-139 | 55244 | 55339 |  |
| DW-EC-140 | 56032 | 56178 |  |
| DW-EC-141 | 56316 | 56474 |  |
| DW-EC-142 | 56855 | 57016 | Hypothetical protein; uncultured Mediterranean phage UvMED-CGR-U-MedDCM-OCT-S44*-C63* |
| DW-EC-143 | 57174 | 57470 | JOODPJME_00081 hypothetical protein \| JIHLJMCN_00141 hypothetical protein \| OAGBNOCD_00094 hypothetical protein \| HLAHOEIE_00186 hypothetical protein \| BDMKCPGI_00141 hypothetical protein \| hypothetical protein; *Escherichia* phage phAPEC8 , complete  genome \| hypothetical protein ECGD1_123; *Enterobacteria* phage ECGD1 \| Phi92_gp115; *Enterobacteria* phage phi92 |
| DW-EC-144 | 57484 | 57627 | Conserved hypothetical protein; *Aeromonas* phage PX29 |
| DW-EC-145 | 57612 | 57782 | DNHOGCFM_00151 hypothetical protein \| hypothetical protein phAPEC8_00144; *Escherichia* phage phAPEC8 \| BDMKCPGI_00142 hypothetical protein \| IFPLOHOB_00141 hypothetical protein \| JOODPJME_00080 hypothetical protein \| hypothetical protein; *Escherichia* phage phAPEC8 \| hypothetical protein phAPEC8_00144; *Escherichia* phage phAPEC8 |
| DW-EC-146 | 57760 | 57849 | JIHLJMCN_00143 hypothetical protein \| BDMKCPGI_00143 hypothetical protein \| Phage protein; ACLAME_Phage_proteins_with_unknown_functions Phage_cyanophage Phage_experimental; *Enterobacteria* phage phi92 \| phage tail sheath protein; uncultured Mediterranean phage uvMED-GF-U-MedDCM-OCT-S28-C30 clone \| baseplate wedge subunit; *Edwardsiella* phage pei26 \| baseplate wedge subunit; *Edwardsiella* phage pei20 |
| DW-EC-147 | 57873 | 58040 |  |
| DW-EC-148 | 58116 | 58460 | Hypothetical protein phAPEC8_00146; *Escherichia* phage phAPEC8 \| HFBDACEP_00145 hypothetical protein \| DNHOGCFM_00153 hypothetical protein \| JOODPJME_00078 hypothetical protein \| JIHLJMCN_00145 hypothetical protein \| hypothetical protein; *Escherichia* phage phAPEC8 |
| DW-EC-149 | 58470 | 60545 | OAGBNOCD_00098 hypothetical protein \| BDMKCPGI_00146 hypothetical protein \| JOODPJME_00077 hypothetical protein \| JIHLJMCN_00146 hypothetical protein \| HLAHOEIE_00190 hypothetical protein \| putative terminase; *Escherichia* phage phAPEC8 \| putative terminase large subunit; *Escherichia* phage phAPEC8 |
| DW-EC-150 | 60647 | 62212 | JOODPJME_00076; hypothetical proteinjihljmcn_00147 hypothetical protein \| OAGBNOCD_00099 hypothetical protein \| HLAHOEIE_00191 hypothetical proteinhfbdacep_00147 hypothetical protein \| hypothetical protein; *Escherichia* phage phAPEC8 \| hypothetical protein phAPEC8_00148' *Escherichia* phage phAPEC8 |
| DW-EC-151 | 62306 | 62785 | JOODPJME_00075 hypothetical protein \| JIHLJMCN_00148 hypothetical protein \| OAGBNOCD_00100 hypothetical protein \| HLAHOEIE_00192 hypothetical protein \| BDMKCPGI_00148 hypothetical protein \| hypothetical protein; *Escherichia* phage phAPEC8 |
| DW-EC-152 | 62788 | 63903 | JOODPJME_00074 hypothetical protein \| JIHLJMCN_00149 hypothetical protein \| OAGBNOCD_00101 hypothetical protein \| DNHOGCFM_00157 hypothetical protein \| hypothetical protein phAPEC8_00150; *Escherichia* phage phAPEC8 \| hypothetical protein; *Escherichia* phage phAPEC8 |
| DW-EC-153 | 63923 | 64321 | JOODPJME_00073 hypothetical protein \| JIHLJMCN_00150 hypothetical protein \| OAGBNOCD_00102 hypothetical protein \| HLAHOEIE_00194 hypothetical protein \| BDMKCPGI_00150 hypothetical protein \| putative head stabilization/decoration protein; *Escherichia* phage phAPEC8 \| putative head stabilization/decoration protein; *Escherichia* phage phAPEC8 |
| DW-EC-154 | 64343 | 64321 | OAGBNOCD_00103 hypothetical protein \| DNHOGCFM_00159 hypothetical protein \| putative major head protein; *Escherichia* phage phAPEC8 \| JOODPJME_00072 hypothetical protein \| JIHLJMCN_00151 hypothetical protein \| putative major head protein; *Escherichia* phage phAPEC8 \| elements of external origin phage-related functions and prophages; *Enterobacteria* phage phi92 \| putative major head protein; *Escherichia* phage phAPEC8 \| major capsid protein; *Enterobacteria* phage ECGD1 \| Phi92_gp124; *Enterobacteria* phage phi92 |
| DW-EC-155 | 65429 | 65773 | Hypothetical protein phAPEC8_00153; *Escherichia* phage phAPEC8 \| BDMKCPGI_00152 hypothetical protein \| JOODPJME_00071 hypothetical protein \| JIHLJMCN_00152 hypothetical protein \| OAGBNOCD_00104 hypothetical protein \| hypothetical protein; *Escherichia* phage phAPEC8 |
| DW-EC-156 | 65783 | 66331 | JOODPJME_00070 hypothetical protein \| OAGBNOCD_00105 hypothetical protein \| HLAHOEIE_00197 hypothetical protein \| DNHOGCFM_00161 hypothetical protein \| hypothetical protein phAPEC8_00154; *Escherichia* phage phAPEC8 \| hypothetical protein; *Escherichia* phage phAPEC8 \| hypothetical protein phAPEC8_00154; *Escherichia* phage phAPEC8 |
| DW-EC-157 | 66331 | 66819 | OAGBNOCD_00106 hypothetical protein \| HFBDACEP_00154 hypothetical protein \| JOODPJME_00069 hypothetical protein \| JIHLJMCN_00154 hypothetical protein \| BDMKCPGI_00154 hypothetical protein \| hypothetical protein; *Escherichia* phage phAPEC8 \| hypothetical protein phAPEC8_00155; *Escherichia* phage phAPEC8 |
| DW-EC-158 | 66841 | 67281 | JIHLJMCN_00155 hypothetical protein \| OAGBNOCD_00107 hypothetical protein \| HFBDACEP_00155 hypothetical protein \| DNHOGCFM_00163 hypothetical protein \| IFPLOHOB_00153 hypothetical protein \| hypothetical protein; *Escherichia* phage phAPEC8 \| hypothetical protein phAPEC8_00156; *Escherichia* phage phAPEC8 |
| DW-EC-159 | 67253 | 67912 | JOODPJME_00067 hypothetical protein \| JIHLJMCN_00156 hypothetical protein \| OAGBNOCD_00108 hypothetical protein \| HLAHOEIE_00200 hypothetical protein \| BDMKCPGI_00156 hypothetical protein \| hypothetical protein; *Escherichia* phage phAPEC8 \| hypothetical protein phAPEC8_00157; *Escherichia* phage phAPEC8 |
| DW-EC-160 | 67947 | 69320 | JOODPJME_00066 hypothetical protein \| JIHLJMCN_00157 hypothetical protein \| OAGBNOCD_00109 hypothetical protein \| HLAHOEIE_00201 hypothetical protein \| HFBDACEP_00157 hypothetical protein \| putative structural protein; *Escherichia* phage phAPEC8 \| putative structural protein; *Escherichia* phage phAPEC8 |
| DW-EC-161 | 69367 | 69846 | JOODPJME_00065 hypothetical proteinjihljmcn_00158 hypothetical protein \| OAGBNOCD_00110 hypothetical protein \| HLAHOEIE_00202 hypothetical protein \| BDMKCPGI_00158 hypothetical protein \| putative structural protein; *Escherichia* phage phAPEC8 \| Phage protein; ACLAME_Phage_proteins_with_unknown_functions  Phage_cyanophage Phage_experimental; *Enterobacteria* phage  phi92 \| putative structural protein; *Escherichia* phage phAPEC8 \| putative tail tube; *Enterobacteria* phage ECGD1 \| Phi92_gp131; *Enterobacteria* phage phi92 |
| DW-EC-162 | 69892 | 70374 | JOODPJME_00064 hypothetical protein \| JIHLJMCN_00159 hypothetical protein \| OAGBNOCD_00111 hypothetical protein \| HLAHOEIE_00203 hypothetical protein \| BDMKCPGI_00159 hypothetical protein \| hypothetical protein; *Escherichia* phage phAPEC8 |
| DW-EC-163 | 70422 | 70640 | Hypothetical protein phAPEC8_00161; *Escherichia* phage phAPEC8 \| hypothetical protein; *Escherichia* phage phAPEC8 \| Alkaline phosphatase, tissue-nonspecific isozyme; Felis catus \| Phi92_gp133 ; *Enterobacteria* phage phi92 |
| DW-EC-164 | 70787 | 72649 | JOODPJME_00063 hypothetical protein \| DNHOGCFM_00168 hypothetical protein \| hypothetical protein phAPEC8_00162; *Escherichia* phage phAPEC8 \| JIHLJMCN_00160 hypothetical protein \| HFBDACEP_00160 hypothetical protein \| hypothetical protein; *Escherichia* phage phAPEC8 |
| DW-EC-165 | 72642 | 72788 |  |
| DW-EC-166 | 72776 | 73477 | DNHOGCFM_00169 hypothetical protein \| hypothetical protein phAPEC8_00163; *Escherichia* phage phAPEC8 \| JOODPJME_00062 hypothetical protein \| JIHLJMCN_00161 hypothetical protein \| BDMKCPGI_00161 hypothetical protein \| hypothetical protein; *Escherichia* phage phAPEC8 \| hypothetical protein phAPEC8_00163; *Escherichia* phage phAPEC8 |
| DW-EC-167 | 73480 | 73893 | JOODPJME_00061 hypothetical protein \| JIHLJMCN_00162 hypothetical proteinbdmkcpgi_00162 hypothetical proteinhfbdacep_00162 hypothetical protein \| DNHOGCFM_00170 hypothetical protein \| hypothetical protein; *Escherichia* phage phAPEC8 \| hypothetical protein phAPEC8_00164; *Escherichia* phage phAPEC8 |
| DW-EC-168 | 73903 | 74913 | HLAHOEIE_00207 hypothetical proteinbdmkcpgi_00163 hypothetical protein \| JOODPJME_00060 hypothetical protein \| JIHLJMCN_00163 hypothetical protein \| HFBDACEP_00163 hypothetical protein \| hypothetical protein; *Escherichia* phage phAPEC8 \| hypothetical protein phAPEC8_00165; *Escherichia* phage phAPEC8 |
| DW-EC-169 | 74913 | 75647 | OAGBNOCD_00116 hypothetical protein \| DNHOGCFM_00172 hypothetical protein \| hypothetical protein phAPEC8_00166; *Escherichia* phage phAPEC8 \| JIHLJMCN_00164 hypothetical protein \| BDMKCPGI_00164 hypothetical protein \| hypothetical protein; *Escherichia* phage phAPEC8 \| hypothetical protein phAPEC8_00166; *Escherichia* phage phAPEC8 |
| DW-EC-170 | 75649 | 76275 | OAGBNOCD_00117 hypothetical protein \| JOODPJME_00058 hypothetical protein \| DNHOGCFM_00173 hypothetical protein \| hypothetical protein phAPEC8_00167; *Escherichia* phage phAPEC8 \| JIHLJMCN_00165 hypothetical protein \| hypothetical protein; *Escherichia* phage phAPEC8 \| hypothetical protein phAPEC8_00167; *Escherichia* phage phAPEC8 |
| DW-EC-171 | 76275 | 76748 | HLAHOEIE_00210 hypothetical protein \| JOODPJME_00057 hypothetical protein \| OAGBNOCD_00118 hypothetical protein \| DNHOGCFM_00174 hypothetical protein \| hypothetical protein phAPEC8_00168; *Escherichia* phage phAPEC8 \| hypothetical protein; *Escherichia* phage phAPEC8 \| hypothetical protein phAPEC8_00168; *Escherichia* phage phAPEC8 |
| DW-EC-172 | 76748 | 79624 | DNHOGCFM_00175 hypothetical protein \| putative structural protein; *Escherichia* phage phAPEC8 \| JIHLJMCN_00167 hypothetical protein \| HFBDACEP_00167 hypothetical proteinhlahoeie_00211 hypothetical protein \| putative structural; *Escherichia* phage phAPEC8 \| putative structural protein; *Escherichia* phage phAPEC8 |
| DW-EC-173 | 79633 | 81786 | HLAHOEIE_00212 hypothetical protein \| DNHOGCFM_00176 hypothetical protein \| hypothetical protein phAPEC8_00170; *Escherichia* phage phAPEC8 \| OAGBNOCD_00120 hypothetical protein \| JOODPJME_00055 hypothetical protein \| hypothetical protein; *Escherichia* phage phAPEC8 |
| DW-EC-174 | 81795 | 83693 | PDNLIHIO_00151 hypothetical protein \| OAGBNOCD_00121 hypothetical protein \| JHACGPAA_00171 hypothetical protein \| DCKEIKPP_00109 hypothetical protein \| JLLHFBGM_00154 hypothetical protein \| tail protein; *Escherichia* phage vb_ecom-ep3 \| tail protein; *Escherichia* phage vb_ecom-ep3 \| tail fiber protein; *Escherichia* phage phAPEC8 \| Phi92_gp143; *Enterobacteria* phage phi92endo-N-acetylneuraminidase or endo-sialidase (EC 3.2.1.129) \| endo-N-acetylneuraminidase or endo-sialidase (EC 3.2.1.129) \| endo-N-acetylneuraminidase or endo-sialidase (EC 3.2.1.129) |
| DW-EC-175 | 83771 | 84079 | HLAHOEIE_00214 hypothetical protein \| JOODPJME_00053 hypothetical protein \| DCKEIKPP_00108 hypothetical protein \| JLLHFBGM_00155 hypothetical protein \| OAGBNOCD_00124 hypothetical protein \| hypothetical protein; *Shigella* phage pSb-1tailspike protein; Salmonella phage vB_SalM_SJ3 \| tailspike protein; Salmonella phage  FSL SP-076 \| Endo-N-acetylneuraminidase (EC 3.2.1.129) (Endo-N) (Endosialidase); *Escherichia* phage vB_EcoM _CBA120 \| Phage tail fiber; ACLAME_Phage_tail  Phage_tail_proteins; *Salmonella* phage SFP10 \| putative tail fiber protein; *Escherichia* phage vB_EcoP_PhAPEC7 \| tail spike protein; *Escherichia* phage Bp4 \| hypothetical protein ECBP1_0072; *Escherichia* phage ECBP1 \| hypothetical protein; *Escherichia* phage EC1-UPM \| hypothetical protein PhAPEC5_67; *Escherichia*  phage phage vB_EcoP_PhAPEC5 \| endorhamnosidase (EC 3.2.1.-) |
| DW-EC-176 | 84076 | 84318 | JOODPJME_00052 hypothetical protein \| HLAHOEIE_00215 hypothetical protein \| OAGBNOCD_00125 hypothetical protein \| DNHOGCFM_00178 hypothetical protein \| hypothetical protein phAPEC8_00172; *Escherichia* phage phAPEC8 \| hypothetical protein; *Escherichia* phage phAPEC8 \| Phage protein; ACLAME_Phage_proteins_with_unknown_functions Phage_cyanophage  Phage_experimental; *Enterobacteria* phage phi92 \| hypothetical protein phAPEC8_00172; *Escherichia* phage phAPEC8 \| Phi92_gp144; *Enterobacteria* phage phi92 \| structural protein; *Enterobacteria* phage ECGD1 |
| DW-EC-177 | 84318 | 85805 | JOODPJME_00051 hypothetical protein \| JIHLJMCN_00170 hypothetical protein \| HLAHOEIE_00216 hypothetical protein \| IFPLOHOB_00168 hypothetical protein \| HFBDACEP_00170 hypothetical protein \| hypothetical protein; *Escherichia* phage phAPEC8 \| hypothetical protein phAPEC8_00173; *Escherichia* phage phAPEC8 |
| DW-EC-178 | 85808 | 86437 | JOODPJME_00050 hypothetical protein \| JIHLJMCN_00171 hypothetical protein \| OAGBNOCD_00127 hypothetical protein \| HLAHOEIE_00217 hypothetical protein \| HFBDACEP_00171 hypothetical protein \| hypothetical protein; phage phAPEC8 \| hypothetical protein phAPEC8_00174; *Escherichia* phage phAPEC8 |
| DW-EC-179 | 86447 | 87508 | JIHLJMCN_00172 hypothetical proteinhfbdacep_00172 hypothetical protein \| IFPLOHOB_00170 hypothetical protein \| DNHOGCFM_00181 hypothetical protein \| putative gph domain protein; *Escherichia* phage phAPEC8 \| putative gph domain protein; *Escherichia* phage phAPEC8 |
| DW-EC-180 | 87509 | 88054 | JOODPJME_00048 hypothetical protein \| HFBDACEP_00173 hypothetical protein \| DNHOGCFM_00182 hypothetical protein \| putative tail fiber assembly protein; *Escherichia* phage phAPEC8 \| JIHLJMCN_00173 hypothetical protein \| putative tail fiber assembly protein; *Escherichia* phage  phAPEC8 \| putative tail fiber assembly protein; *Escherichia* phage phAPEC8 |
| DW-EC-181 | 88064 | 88399 | JOODPJME_00047 hypothetical protein \| JIHLJMCN_00174 hypothetical protein \| OAGBNOCD_00130 hypothetical protein \| HLAHOEIE_00220 hypothetical protein \| BDMKCPGI_00175 hypothetical protein \| hypothetical protein; *Escherichia* phage phAPEC8 |
| DW-EC-182 | 88403 | 91456 | OAGBNOCD_00131 hypothetical protein \| JOODPJME_00046 hypothetical protein \| DNHOGCFM_00184 hypothetical protein \| IFPLOHOB_00173 hypothetical proteinputative colanic acid-degrading protein; *Escherichia* phage phAPEC8 \| putative colanic acid-degrading protein; *Escherichia* phage phAPEC8 \| putative colanic acid-degrading protein; *Escherichia* phage phAPEC8 |
| DW-EC-183 | 91518 | 93542 | DNHOGCFM_00185 hypothetical protein \| putative phage tail fiber protein; *Escherichia* phage phAPEC8 \| JIHLJMCN_00176 hypothetical protein \| OAGBNOCD_00132 hypothetical protein \| BDMKCPGI_00177 hypothetical protein \| putative phage tail fiber protein; *Escherichia* phage phAPEC8 |
| DW-EC-184 | 93584 | 96256 | BDMKCPGI_00178 hypothetical protein \| HLAHOEIE_00223 hypothetical protein \| HFBDACEP_00177 hypothetical protein \| DNHOGCFM_00186 hypothetical protein \| hypothetical protein phAPEC8_00180; *Escherichia* phage phAPEC8 \| hypothetical protein; *Escherichia* phage phAPEC8 |
| DW-EC-185 | 96361 | 97248 | JOODPJME_00043 hypothetical protein \| JIHLJMCN_00179 hypothetical protein \| HFBDACEP_00178 hypothetical protein \| IFPLOHOB_00176 hypothetical protein \| HLAHOEIE_00224 hypothetical protein \| hypothetical protein; *Escherichia* phage phAPEC8 \| Single-stranded DNA-binding protein 1; *Salmonella typhimurium* (strain LT2 / SGSC1412 / ATCC 700720)Single-stranded DNA-binding protein 1; *Salmonella typhi* O \| Single-stranded DNA-binding protein; Shigella flexneri \| hypothetical protein ECGD1_164; *Enterobacteria* phage ECGD1 \| Phi92_gp156; *Enterobacteria* phage phi92 \| Single-stranded DNA-binding protein; *Escherichia* phage RCS47 \| single-stranded DNA-binding protein; *Vibrio* phage pyd38-A |
| DW-EC-186 | 97265 | 97657 | JOODPJME_00042 hypothetical protein \| JIHLJMCN_00180 hypothetical protein \| HLAHOEIE_00225 hypothetical protein \| HFBDACEP_00179 hypothetical protein \| DNHOGCFM_00188 hypothetical protein \| hypothetical protein; *Escherichia* phage phAPEC8 |
| DW-EC-187 | 97703 | 98296 | JIHLJMCN_00181 hypothetical protein \| DNHOGCFM_00189 hypothetical protein \| putative tRNA nucleotidyl transferase / poly(A) polymerase; *Escherichia* phage phAPEC8 \| HLAHOEIE_00226 hypothetical protein \| JOODPJME_00041 hypothetical protein \| putative tRNA nucleotidyl transferase / poly(A) polymerase; *Escherichia* phage phAPEC8 \| putative tRNA nucleotidyl transferase / poly(A) polymerase; *Escherichia* phage phAPEC8 |
| DW-EC-188 | 98289 | 98507 | JIHLJMCN_00182 hypothetical protein \| DNHOGCFM_00190 hypothetical protein \| hypothetical protein phAPEC8_00184; *Escherichia* phage phAPEC8 \| JOODPJME_00040 hypothetical protein \| OAGBNOCD_00138 hypothetical protein \| hypothetical protein; *Escherichia* phage phAPEC8 |
| DW-EC-189 | 98518 | 98685 | BDMKCPGI_00183 hypothetical protein \| JIHLJMCN_00183 hypothetical protein \| DNHOGCFM_00191 hypothetical protein \| hypothetical protein phAPEC8_00185; *Escherichia* phage phAPEC8 \| HLAHOEIE_00228 hypothetical protein \| hypothetical protein; *Escherichia* phage phAPEC8 |
| DW-EC-190 | 98685 | 98876 | JIHLJMCN_00184 hypothetical protein \| HLAHOEIE_00229 hypothetical protein \| DNHOGCFM_00192 hypothetical protein \| hypothetical protein phAPEC8_00186; *Escherichia* phage phAPEC8 \| BDMKCPGI_00184 hypothetical protein \| hypothetical protein; *Escherichia* phage phAPEC8 |
| DW-EC-191 | 98926 | 101523 | JIHLJMCN_00185 hypothetical protein \| HLAHOEIE_00230 hypothetical protein \| DNHOGCFM_00193 hypothetical protein \| putative DNA polymerase/3'-5' exonuclease domain; *Escherichia* phage phAPEC8 \| IFPLOHOB_00182 hypothetical protein \| putative DNA polymerase/3'-5' exonuclease domain; *Escherichia* phage phAPEC8 \| putative DNA polymerase/3'-5' exonuclease domain; *Escherichia* phage phAPEC8 |
| DW-EC-192 | 101523 | 102317 | JIHLJMCN_00186 hypothetical protein \| OAGBNOCD_00141 hypothetical protein \| HLAHOEIE_00231 hypothetical protein \| BDMKCPGI_00186 hypothetical proteinhfbdacep_00185 hypothetical protein \| putative DNA N6-adenine methyltransferase; *Escherichia* phage phAPEC8 \| putative DNA N6-adenine methyltransferase; *Escherichia* phage phAPEC8 |
| DW-EC-193 | 102327 | 102758 | JOODPJME_00035 hypothetical protein \| JIHLJMCN_00187 hypothetical protein \| OAGBNOCD_00142 hypothetical protein \| HLAHOEIE_00232 hypothetical protein \| BDMKCPGI_00187 hypothetical protein \| hypothetical protein; *Escherichia* phage phAPEC8 |
| DW-EC-194 | 102851 | 104629 | JOODPJME_00034 hypothetical protein \| DNHOGCFM_00196 hypothetical protein \| IFPLOHOB_00185 hypothetical protein \| putative primase/helicase; *Escherichia* phage phAPEC8 \| JIHLJMCN_00188 hypothetical protein \| putative primase/helicase; *Escherichia* phage phAPEC8 |
| DW-EC-195 | 104642 | 104920 | JOODPJME_00033 hypothetical protein \| JIHLJMCN_00189 hypothetical protein \| OAGBNOCD_00144 hypothetical protein \| HLAHOEIE_00234 hypothetical protein \| BDMKCPGI_00189 hypothetical protein \| hypothetical protein; *Escherichia* phage phAPEC8 |
| DW-EC-196 | 104920 | 105864 | OAGBNOCD_00145 hypothetical protein \| DNHOGCFM_00198 hypothetical protein \| hypothetical protein phAPEC8_00192; *Escherichia* phage phAPEC8 \| IFPLOHOB_00187 hypothetical proteinhlahoeie_00235 hypothetical protein \| hypothetical protein; *Escherichia* phage phAPEC8 \| sucrose synthase (EC 2.4.1.13) \| sucrose synthase (EC 2.4.1.13) \| sucrose synthase (EC 2.4.1.13) \| sucrose synthase (EC 2.4.1.13) \| sucrose synthase (EC 2.4.1.13) |
| DW-EC-197 | 105872 | 106819 | JOODPJME_00031 hypothetical protein \| HLAHOEIE_00236 hypothetical protein \| BDMKCPGI_00191 hypothetical protein \| OAGBNOCD_00146 hypothetical protein \| IFPLOHOB_00188 hypothetical protein \| hypothetical protein; *Escherichia* phage phAPEC8 \| sucrose synthase (EC 2.4.1.13) \| sucrose synthase (EC 2.4.1.13) \| sucrose synthase (EC 2.4.1.13) \| sucrose synthase (EC 2.4.1.13) \| sucrose synthase (EC 2.4.1.13) |
| DW-EC-198 | 106809 | 107492 | DNHOGCFM_00200 hypothetical protein \| hypothetical protein phAPEC8_00194; *Escherichia* phage phAPEC8 \| OAGBNOCD_00147 hypothetical protein \| JIHLJMCN_00192 hypothetical protein \| HFBDACEP_00191 hypothetical protein \| hypothetical protein; *Escherichia* phage phAPEC8 |
| DW-EC-199 | 107501 | 108064 | JOODPJME_00029 hypothetical protein \| JIHLJMCN_00193 hypothetical protein \| OAGBNOCD_00148 hypothetical protein \| HLAHOEIE_00238 hypothetical protein \| BDMKCPGI_00193 hypothetical protein \| hypothetical protein; *Escherichia* phage phAPEC8 |
| DW-EC-200 | 108081 | 108272 | JOODPJME_00028 hypothetical protein \| JIHLJMCN_00194 hypothetical proteinoagbnocd_00149 hypothetical protein \| HLAHOEIE_00239 hypothetical protein \| BDMKCPGI_00194 hypothetical protein \| hypothetical protein; *Escherichia* phage phAPEC8 \| hypothetical protein phAPEC8_00196; *Escherichia* phage phAPEC8 |
| DW-EC-201 | 108250 | 108528 | JOODPJME_00027 hypothetical protein \| JIHLJMCN_00195 hypothetical protein \| OAGBNOCD_00150 hypothetical protein \| HLAHOEIE_00240 hypothetical protein \| BDMKCPGI_00195 hypothetical protein \| hypothetical protein; *Escherichia* phage phAPEC8 |
| DW-EC-202 | 108480 | 108794 | JIHLJMCN_00196 hypothetical protein \| OAGBNOCD_00151 hypothetical protein \| HLAHOEIE_00241 hypothetical protein \| HFBDACEP_00195 hypothetical protein \| DNHOGCFM_00204 hypothetical protein \| hypothetical protein; *Escherichia* phage phAPEC8 \| hypothetical protein ECGD1_178; *Enterobacteria* phage ECGD1 \| Phi92_gp169; *Enterobacteria* phage phi92 |
| DW-EC-203 | 108766 | 109287 | HLAHOEIE_00242 hypothetical protein \| DNHOGCFM_00205 hypothetical protein \| putative cell wall hydrolase SleB; *Escherichia* phage phAPEC8 \| OAGBNOCD_00152 hypothetical protein \| JOODPJME_00025 hypothetical protein \| putative cell wall hydrolase sleb; *Escherichia* phage phAPEC8 \| putative cell wall hydrolase SleB; *Escherichia* phage phAPEC8 |
| DW-EC-204 | 109387 | 109989 | JOODPJME_00024 General stress protein 16U \| JIHLJMCN_00198 General stress protein 16U \| OAGBNOCD_00153 General stress protein 16U \| HLAHOEIE_00243 General stress protein 16U \| BDMKCPGI_00198 General stress protein 16U \| hypothetical protein; *Escherichia* phage phAPEC8 \| hypothetical protein phAPEC8_00200; *Escherichia* phage phAPEC8 |
| DW-EC-205 | 110005 | 110274 | JOODPJME_00023 hypothetical protein \| JIHLJMCN_00199 hypothetical protein \| OAGBNOCD_00154 hypothetical protein \| BDMKCPGI_00199 hypothetical protein \| HFBDACEP_00198 hypothetical protein \| hypothetical protein; *Escherichia* phage phAPEC8 |
| DW-EC-206 | 110255 | 110503 | JOODPJME_00022 hypothetical protein \| JIHLJMCN_00200 hypothetical protein \| IFPLOHOB_00197 hypothetical protein \| OAGBNOCD_00155 hypothetical protein \| HLAHOEIE_00245 hypothetical protein \| hypothetical protein; *Escherichia* phage phAPEC8 |
| DW-EC-207 | 110500 | 111294 | JOODPJME_00021 hypothetical protein \| JIHLJMCN_00201 hypothetical protein \| HFBDACEP_00200 hypothetical protein \| OAGBNOCD_00156 hypothetical protein \| BDMKCPGI_00201 hypothetical protein \| hypothetical protein; *Escherichia* phage phAPEC8 \| hypothetical protein phAPEC8_00203; *Escherichia* phage phAPEC8 |
| DW-EC-208 | 111296 | 111574 | JOODPJME_00020 hypothetical protein \| OAGBNOCD_00157 hypothetical protein \| HLAHOEIE_00247 hypothetical protein \| HFBDACEP_00201 hypothetical protein \| DNHOGCFM_00210 hypothetical protein \| hypothetical protein; *Escherichia* phage phAPEC8 |
| DW-EC-209 | 111567 | 112130 | DNHOGCFM_00211 hypothetical protein \| hypothetical protein phAPEC8_00205; *Escherichia* phage phAPEC8 \| HLAHOEIE_00248 hypothetical protein \| JOODPJME_00019 hypothetical protein \| JIHLJMCN_00203 hypothetical protein \| hypothetical protein; *Escherichia* phage phAPEC8 |
| DW-EC-210 | 112130 | 112528 | DNHOGCFM_00212 hypothetical protein \| hypothetical protein phAPEC8_00206; *Escherichia* phage phAPEC8 \| IFPLOHOB_00201 hypothetical protein \| JOODPJME_00018 hypothetical protein \| JIHLJMCN_00204 hypothetical protein \| hypothetical protein; *Escherichia* phage phAPEC8 |
| DW-EC-211 | 112521 | 112694 | JOODPJME_00017 hypothetical proteinjihljmcn_00205 hypothetical protein \| OAGBNOCD_00160 hypothetical protein \| BDMKCPGI_00205 hypothetical proteinhfbdacep_00204 hypothetical proteinhypothetical protein; *Escherichia* phage phAPEC8 \| hypothetical protein phAPEC8_00207; *Escherichia* phage phAPEC8 |
| DW-EC-212 | 112694 | 112891 | HLAHOEIE_00251 hypothetical protein \| BDMKCPGI_00206 hypothetical proteinjihljmcn_00206 hypothetical protein \| HFBDACEP_00205 hypothetical proteindnhogcfm_00214 hypothetical protein \| hypothetical protein; *Escherichia* phage phAPEC8 |
| DW-EC-213 | 112888 | 114000 | OAGBNOCD_00162 TelA-like protein \| BDMKCPGI_00207 TelA -like protein \| IFPLOHOB_00204 TelA -like protein \| JOODPJME_00015 TelA -like protein \| JIHLJMCN_00207 TelA -like protein \| hypothetical protein; *Escherichia* phage phAPEC8 |
| DW-EC-214 | 113997 | 114587 | HLAHOEIE_00253 hypothetical protein \| IFPLOHOB_00205 hypothetical protein \| hypothetical protein phAPEC8_00210; *Escherichia* phage phAPEC8 \| JOODPJME_00014 hypothetical protein \| JIHLJMCN_00208 hypothetical protein \| hypothetical protein; *Escherichia* phage phAPEC8 \| hypothetical protein phAPEC8_00210; *Escherichia* phage phAPEC8 |
| DW-EC-215 | 114590 | 115183 | HLAHOEIE_00254 hypothetical protein \| BDMKCPGI_00209 hypothetical protein \| OAGBNOCD_00164 hypothetical protein \| DNHOGCFM_00217 hypothetical protein \| hypothetical protein phAPEC8_00211; *Escherichia* phage phAPEC8 \| hypothetical protein; *Escherichia* phage phAPEC8 |
| DW-EC-216 | 115198 | 115710 | Hypothetical protein phAPEC8_00212; *Escherichia* phage phAPEC8 \| DNHOGCFM_00218 hypothetical protein \| OAGBNOCD_00165 hypothetical protein \| JOODPJME_00012 hypothetical protein \| JIHLJMCN_00210 hypothetical protein \| hypothetical protein; *Escherichia* phage phAPEC8 |
| DW-EC-217 | 115819 | 116169 | HLAHOEIE_00257 hypothetical protein \| IFPLOHOB_00209 hypothetical protein \| BDMKCPGI_00212 hypothetical protein \| DNHOGCFM_00220 hypothetical protein \| hypothetical protein phAPEC8_00213; *Escherichia* phage phAPEC8 \| hypothetical protein; *Escherichia* phage phAPEC8 |
| DW-EC-218 | 116212 | 117186 | OAGBNOCD_00168 Putative membrane-bound redox modulator Alx \| DNHOGCFM_00221 Putative membrane-bound redox modulator Alx \| integral membrane protein terc; *Escherichia* phage phAPEC8 \| HLAHOEIE_00258 Putative membrane-bound redox modulator Alx \| JIHLJMCN_00213 Putative membrane-bound redox modulator Alx \| integral membrane protein terc; *Escherichia* phage phAPEC8 \| integral membrane protein terc; *Escherichia* phage phAPEC8 |
| DW-EC-219 | 117266 | 117487 | JOODPJME_00008 hypothetical protein \| OAGBNOCD_00169 hypothetical protein \| HLAHOEIE_00259 hypothetical protein \| HFBDACEP_00213 hypothetical protein \| IFPLOHOB_00211 hypothetical protein \| hypothetical protein; *Escherichia* phage phAPEC8 |
| DW-EC-220 | 117500 | 117973 | DNHOGCFM_00223 hypothetical protein \| hypothetical protein phAPEC8_00216; *Escherichia* phage phAPEC8 \| JOODPJME_00007 hypothetical protein \| BDMKCPGI_00215 hypothetical protein \| JIHLJMCN_00215 hypothetical protein \| hypothetical protein; *Escherichia* phage phAPEC8 |
| DW-EC-221 | 117987 | 118814 | OAGBNOCD_00171 hypothetical protein \| DNHOGCFM_00224 hypothetical protein \| hypothetical protein phAPEC8_00217; *Escherichia* phage phAPEC8 \| JOODPJME_00006 hypothetical protein \| JIHLJMCN_00216 hypothetical protein \| hypothetical protein; *Escherichia* phage phAPEC8 c8 \| hypothetical protein phAPEC8_00217; *Escherichia* phage phAPEC8 |
| DW-EC-222 | 118807 | 119463 | DNHOGCFM_00225 hypothetical protein \| hypothetical protein phAPEC8_00218; *Escherichia* phage phAPEC8 \| JOODPJME_00005 hypothetical protein \| HLAHOEIE_00262 hypothetical protein \| hypothetical protein; *Escherichia* phage phAPEC8 \| hypothetical protein phAPEC8_00218; *Escherichia* phage phAPEC8 |
| DW-EC-223 | 119465 | 120070 | JOODPJME_00004 hypothetical protein \| OAGBNOCD_00173 hypothetical protein \| HFBDACEP_00217 hypothetical protein \| DNHOGCFM_00226 hypothetical protein \| hypothetical protein phAPEC8_00219; *Escherichia* phage phAPEC8 \| hypothetical protein; *Escherichia* phage phAPEC8 |
| DW-EC-224 | 120086 | 120277 | JOODPJME_00003 hypothetical protein \| OAGBNOCD_00174 hypothetical protein \| BDMKCPGI_00219 hypothetical protein \| HFBDACEP_00218 hypothetical protein \| DNHOGCFM_00227 hypothetical protein \| hypothetical protein; *Escherichia* phage phAPEC8 |
| DW-EC-225 | 120291 | 120761 | DNHOGCFM_00228 Anaerobic ribonucleoside-triphosphate reductase-activating protein \| ribonucleotide reductase of class III (anaerobic) activating protein; *Escherichia* phage phAPEC8 \| OAGBNOCD_00175 Anaerobic ribonucleoside-triphosphate reductase-activating proteinjoodpjme_00002 Anaerobic ribonucleoside-triphosphate reductase-activating protein \| BDMKCPGI_00220 Anaerobic ribonucleoside-triphosphate reductase-activating protein \| ribonucleotide reductase of class III (anaerobic) activating protein; *Escherichia* phage phAPEC8 |
| DW-EC-226 | 120758 | 122572 | HLAHOEIE_00266 Anaerobic ribonucleoside-triphosphate reductase \| JIHLJMCN_00221 Anaerobic ribonucleoside-triphosphate reductase \| BDMKCPGI_00221 Anaerobic ribonucleoside-triphosphate reductase \| HFBDACEP_00220 Anaerobic ribonucleoside-triphosphate reductase ;OAGBNOCD_00176 Anaerobic ribonucleoside-triphosphate reductase \| ribonucleotide reductase of class III (anaerobic) large subunit; *Escherichia* phage  phAPEC8 \| ribonucleotide reductase of class III (anaerobic) large subunit; *Escherichia* phage phAPEC8 |
| DW-EC-227 | 122673 | 124019 | HLAHOEIE_00267 hypothetical proteinjoodpjme_00283 hypothetical protein \| OAGBNOCD_00177 hypothetical protein |
| DW-EC-228 | 124016 | 124570 | JOODPJME_00282 hypothetical proteinoagbnocd_00178 hypothetical proteinhlahoeie_00268 hypothetical protein \| IFPLOHOB_00220 hypothetical protein \| Phage protein; ACLAME_Phage_proteins_with_unknown_functions Phage_cyanophage  Phage_experimental; *Enterobacteria* phage phi92 \| hypothetical protein; *Escherichia* phage phAPEC8 \| Phi92_gp196; *Enterobacteria* phage phi92 \| putative seryl-tRNA synthetase; *Enterobacteria* phage ECGD1 \| hypothetical protein phAPEC8_00224; *Escherichia* phage phAPEC8 |
| DW-EC-229 | 124567 | 124818 | JOODPJME_00281 hypothetical proteinjihljmcn_00224 hypothetical protein \| HLAHOEIE_00269 hypothetical protein \| BDMKCPGI_00224 hypothetical protein \| HFBDACEP_00223 hypothetical protein \| hypothetical protein; *Escherichia* phage phAPEC8 \| hypothetical protein phAPEC8_00225; *Escherichia* phage phAPEC8 |
| DW-EC-230 | 124819 | 126411 | IFPLOHOB_00222 ATP-dependent DNA helicase Rep \| DNHOGCFM_00233 ATP-dependent DNA helicase Rep \| putative uvrd-type helicase; *Escherichia* phage phAPEC8 \| OAGBNOCD_00180 ATP-dependent DNA helicase Rep \| HLAHOEIE_00270 ATP-dependent DNA helicase Rep \| putative UvrD -type helicase; *Escherichia* phage phAPEC8 \| putative UvrD -type helicase; *Escherichia* phage phAPEC8 |
| DW-EC-231 | 126426 | 126935 | JOODPJME_00279 hypothetical protein \| JIHLJMCN_00226 hypothetical protein \| OAGBNOCD_00181 hypothetical protein \| HLAHOEIE_00271 hypothetical protein \| BDMKCPGI_00226 hypothetical protein \| hypothetical protein; *Escherichia* phage phAPEC8 \| hypothetical protein phAPEC8_00227; *Escherichia* phage phAPEC8 |
| DW-EC-232 | 126949 | 127359 | JOODPJME_00278 hypothetical protein \| JIHLJMCN_00227 hypothetical protein \| OAGBNOCD_00182 hypothetical protein \| HLAHOEIE_00272 hypothetical protein \| BDMKCPGI_00227 hypothetical protein \| hypothetical protein; *Escherichia* phage phAPEC8 \| hypothetical protein phAPEC8_00228; *Escherichia* phage phAPEC8 |
| DW-EC-233 | 127299 | 127667 | JIHLJMCN_00228 hypothetical protein \| BDMKCPGI_00228 hypothetical protein \| HFBDACEP_00227 hypothetical protein \| HLAHOEIE_00273 hypothetical proteindnhogcfm_00236 hypothetical protein \| hypothetical protein; *Escherichia* phage phAPEC8 \| Phage protein; ACLAME_Phage_proteins_with_unknown_functions Phage_cyanophage  Phage_experimental; *Enterobacteria* phage phi92 \| hypothetical protein phAPEC8_00229; *Escherichia* phage phAPEC8 \| putative ribonucleoside-diphosphate reductase 1 alpha subunit; *Enterobacteria*  phage ECGD1 \| Phi92_gp202; *Enterobacteria* phage phi92 |
| DW-EC-234 | 127669 | 128031 | OAGBNOCD_00184 hypothetical protein \| DNHOGCFM_00237 hypothetical protein \| IFPLOHOB_00226 hypothetical protein \| hypothetical protein phAPEC8_00230; *Escherichia* phage phAPEC8 \| JOODPJME_00276 hypothetical protein \| hypothetical protein; *Escherichia* phage phAPEC8 |
| DW-EC-235 | 128028 | 128390 | OAGBNOCD_00185 hypothetical protein \| HLAHOEIE_00275 hypothetical protein |
| DW-EC-236 | 128391 | 128696 | OAGBNOCD_00186 hypothetical protein \| HLAHOEIE_00276 hypothetical protein |
| DW-EC-237 | 128696 | 129004 | JOODPJME_00274 hypothetical protein \| JIHLJMCN_00231 hypothetical protein \| BDMKCPGI_00231 hypothetical proteinhfbdacep_00230 hypothetical protein \| DNHOGCFM_00239 hypothetical protein \| hypothetical protein; *Escherichia* phage phAPEC8 |
| DW-EC-238 | 129004 | 129177 | JOODPJME_00273 hypothetical protein \| JIHLJMCN_00232 hypothetical protein \| HFBDACEP_00231 hypothetical protein \| IFPLOHOB_00229 hypothetical protein \| BDMKCPGI_00232 hypothetical protein \| hypothetical protein; *Escherichia* phage phAPEC8 \| hypothetical protein phAPEC8_00233' *Escherichia* phage phAPEC8 |
| DW-EC-239 | 129221 | 129418 | JOODPJME_00272 hypothetical protein \| JIHLJMCN_00233 hypothetical protein \| OAGBNOCD_00189 hypothetical protein \| HLAHOEIE_00279 hypothetical protein \| BDMKCPGI_00233 hypothetical protein \| hypothetical protein; *Escherichia* phage phAPEC8 \| terminase; *Listeria* phage LMTA-94 \| terminase; *Listeria* phage LMTA-57 \| terminase; *Listeria* phage LMTA-148 \| gp5; *Listeria* virus P100 \| gp74; *Listeria* virus A511 |
| DW-EC-240 | 129501 | 129770 | HLAHOEIE_00280 hypothetical protein \| DNHOGCFM_00242 hypothetical protein \| hypothetical protein phAPEC8_00235; *Escherichia* phage phAPEC8 \| JOODPJME_00271 hypothetical protein \| JIHLJMCN_00234 hypothetical protein \| hypothetical protein; *Escherichia* phage phAPEC8 |
| DW-EC-241 | 129767 | 129964 | HLAHOEIE_00281 hypothetical protein \| DNHOGCFM_00243 hypothetical protein \| hypothetical protein phAPEC8_00236; *Escherichia* phage phAPEC8 \| JOODPJME_00270 hypothetical protein \| JIHLJMCN_00235 hypothetical protein \| hypothetical protein; *Escherichia* phage phAPEC8 |
| DW-EC-242 | 130004 | 130459 | OAGBNOCD_00192 hypothetical protein \| HLAHOEIE_00282 hypothetical protein \| DNHOGCFM_00244 hypothetical protein \| hypothetical protein phAPEC8_00237; *Escherichia* phage phAPEC8 \| JOODPJME_00269 hypothetical protein \| hypothetical protein; *Escherichia* phage phAPEC8 |
| DW-EC-243 | 130452 | 130601 | JOODPJME_00268 hypothetical protein \| JIHLJMCN_00237 hypothetical protein \| HLAHOEIE_00283 hypothetical protein \| BDMKCPGI_00237 hypothetical protein \| HFBDACEP_00236 hypothetical protein \| hypothetical protein; *Escherichia* phage phAPEC8 \| hypothetical protein phAPEC8_00238; *Escherichia* phage phAPEC8 |
| DW-EC-244 | 130661 | 130921 | HLAHOEIE_00284 hypothetical protein \| OAGBNOCD_00194 hypothetical protein \| DNHOGCFM_00246 hypothetical protein \| hypothetical protein phAPEC8_00239; *Escherichia* phage phAPEC8 \| hypothetical protein; *Escherichia* phage phAPEC8 |
| DW-EC-245 | 130918 | 131067 | DNHOGCFM_00247 hypothetical protein \| YP_007348608.1 \| hypothetical protein phAPEC8_00240 ; *Escherichia* phage phAPEC8 \| OAGBNOCD_00195 hypothetical protein \| JOODPJME_00265 hypothetical protein \| JIHLJMCN_00240 hypothetical protein \| hypothetical protein; *Escherichia* phage phAPEC8 \| Phage protein; ACLAME_Phage_proteins_with_unknown_functions Phage_cyanophage  Phage_experimental; *Enterobacteria* phage phi92 \| hypothetical protein phAPEC8_00240; *Escherichia* phage phAPEC8 \| hypothetical protein ECGD1_220; *Enterobacteria* phage ECGD1 \| Phi92_gp213; *Enterobacteria* phage phi92 \| exo-beta-1,3-glucanase (EC 3.2.1.58) |
| DW-EC-246 | 131064 | 131498 | OAGBNOCD_00196 hypothetical protein \| DNHOGCFM_00248 hypothetical protein \| hypothetical protein phAPEC8_00241; *Escherichia* phage phAPEC8 \| JOODPJME_00264 hypothetical protein \| JIHLJMCN_00241 hypothetical protein \| hypothetical protein; *Escherichia* phage phAPEC8 \| hyphothetical protein; *Escherichia* phage 4MG \| hypothetical protein KB57_229; Klebsiella phage vb_kpnm_KB57 |
| DW-EC-247 | 131480 | 131587 | RL1_HALLT 50S ribosomal protein L1; *Halorubrum lacusprofundi* (strain ATCC 49239 / DSM 5036 / JCM 8891 / ACAM 34) |
| DW-EC-248 | 131616 | 131906 | JOODPJME_00263 hypothetical protein \| JIHLJMCN_00242 hypothetical protein \| OAGBNOCD_00197 hypothetical protein \| BDMKCPGI_00242 hypothetical protein \| HFBDACEP_00241 hypothetical protein \| hypothetical protein; *Escherichia* phage phAPEC8 \| hypothetical protein phAPEC8_00242; *Escherichia* phage phAPEC8 |
| DW-EC-249 | 131919 | 132410 | DNHOGCFM_00250 hypothetical protein \| hypothetical protein phAPEC8_00243; *Escherichia* phage phAPEC8 \| hypothetical protein; *Escherichia* phage phAPEC8 \| hypothetical protein phAPEC8_00243; *Escherichia* phage phAPEC8 |
| DW-EC-250 | 132448 | 132735 | DNHOGCFM_00251 hypothetical protein \| hypothetical protein phAPEC8_00244; *Escherichia* phage phAPEC8 \| OAGBNOCD_00198 hypothetical protein \| HLAHOEIE_00002 hypothetical protein \| BDMKCPGI_00243 hypothetical protein \| hypothetical protein; *Escherichia* phage phAPEC8 \| Phage protein; ACLAME_Phage_proteins_with_unknown_functions Phage_cyanophage  Phage_experimental; *Enterobacteria* phage phi92 \| hypothetical protein phAPEC8_00244; *Escherichia* phage phAPEC8 |
| DW-EC-251 | 132732 | 133088 | JOODPJME_00261 hypothetical protein \| JIHLJMCN_00244 hypothetical protein \| HLAHOEIE_00003 hypothetical protein \| BDMKCPGI_00244 hypothetical protein \| HFBDACEP_00243 hypothetical protein \| hypothetical protein; *Escherichia* phage phAPEC8 |
| DW-EC-252 | 133091 | 133267 | OAGBNOCD_00200 hypothetical protein \| BDMKCPGI_00245 hypothetical protein \| IFPLOHOB_00242 hypothetical protein \| JOODPJME_00260 hypothetical protein \| JIHLJMCN_00245 hypothetical protein \| hypothetical protein; *Escherichia* phage phAPEC8 |
| DW-EC-253 | 133278 | 133616 | JOODPJME_00259 hypothetical protein \| JIHLJMCN_00246 hypothetical protein \| OAGBNOCD_00201 hypothetical protein \| HLAHOEIE_00005 hypothetical protein \| BDMKCPGI_00246 hypothetical protein \| hypothetical protein; *Escherichia* phage phAPEC8 \| hypothetical protein phAPEC8_00247; *Escherichia* phage phAPEC8 \| hypothetical protein ECGD1_225; *Enterobacteria* phage ECGD1 |
| DW-EC-254 | 133616 | 133867 | OAGBNOCD_00202 hypothetical protein \| HLAHOEIE_00006 hypothetical protein \| DNHOGCFM_00255 hypothetical protein \| hypothetical protein phAPEC8_00248; *Escherichia* phage phAPEC8 \| JOODPJME_00258 hypothetical protein \| hypothetical protein; *Escherichia* phage phAPEC8 |
| DW-EC-255 | 133881 | 134045 | JOODPJME_00257 hypothetical protein \| JIHLJMCN_00248 hypothetical protein \| OAGBNOCD_00203 hypothetical protein \| BDMKCPGI_00248 hypothetical proteinhfbdacep_00247 hypothetical protein \| hypothetical protein; *Escherichia* phage phAPEC8 |
| DW-EC-256 | 134055 | 134219 | JOODPJME_00256 hypothetical protein \| JIHLJMCN_00249 hypothetical protein \| OAGBNOCD_00204 hypothetical protein \| BDMKCPGI_00249 hypothetical protein \| HFBDACEP_00248 hypothetical protein \| hypothetical protein; *Escherichia* phage phAPEC8 |
| DW-EC-257 | 134231 | 134587 | OAGBNOCD_00205 hypothetical protein \| HLAHOEIE_00009 hypothetical protein \| HFBDACEP_00249 hypothetical protein \| IFPLOHOB_00247 hypothetical protein \| BDMKCPGI_00250 hypothetical protein \| hypothetical protein; *Escherichia* phage phAPEC8 |
| DW-EC-258 | 134587 | 135726 | JOODPJME_00254 hypothetical protein \| JIHLJMCN_00251 hypothetical protein \| OAGBNOCD_00206 hypothetical protein \| HLAHOEIE_00010 hypothetical protein \| BDMKCPGI_00251 hypothetical protein \| putative ATPase; *Escherichia* phage phAPEC8 \| putative ATPase; *Escherichia* phage phAPEC8 |
| DW-EC-259 | 135828 | 137027 | JOODPJME_00253 hypothetical protein \| HLAHOEIE_00011 hypothetical protein \| DNHOGCFM_00260 hypothetical protein \| hypothetical protein phAPEC8_00253; *Escherichia* phage phAPEC8 \| hypothetical protein; *Escherichia* phage phAPEC8 \| hypothetical protein; Yersinia phage phir201 \| hypothetical protein phAPEC8_00253; *Escherichia* phage phAPEC8 \| hypothetical protein BN79_038; *Yersinia* phage phir201 |
| DW-EC-260 | 137021 | 137386 | JOODPJME_00252 hypothetical protein \| JIHLJMCN_00253 hypothetical protein \| OAGBNOCD_00208 hypothetical protein \| HLAHOEIE_00012 hypothetical protein \| BDMKCPGI_00253 hypothetical protein \| hypothetical protein; *Escherichia* phage phAPEC8 |
| DW-EC-261 | 137400 | 137588 | DNHOGCFM_00262 hypothetical protein \| hypothetical protein phAPEC8_00255; *Escherichia* phage phAPEC8 \| OAGBNOCD_00209 hypothetical protein \| JOODPJME_00251 hypothetical protein \| BDMKCPGI_00254 hypothetical protein \| hypothetical protein; *Escherichia* phage phAPEC8 |
| DW-EC-262 | 137575 | 138108 | JOODPJME_00250 hypothetical protein \| JIHLJMCN_00255 hypothetical protein \| HFBDACEP_00254 hypothetical protein \| IFPLOHOB_00252 hypothetical protein \| OAGBNOCD_00210 hypothetical protein \| hypothetical protein; *Escherichia* phage phAPEC8 \| hypothetical protein phAPEC8_00256; *Escherichia* phage phAPEC8 |
| DW-EC-263 | 138149 | 138283 |  |
| DW-EC-264 | 138416 | 138610 |  |
| DW-EC-265 | 138692 | 138844 | Hypothetical protein phAPEC8_00257; *Escherichia* phage phAPEC8 \| hypothetical protein; *Escherichia* phage phAPEC8 \| alpha-mannosidase (EC 3.2.1.113) |
| DW-EC-266 | 138838 | 139032 | Hypothetical protein phAPEC8_00257; *Escherichia* phage phAPEC8 \| hypothetical protein; *Escherichia* phage phAPEC8 \| hypothetical protein Sano_37; *Xylella* phage Sano |
| DW-EC-267 | 139099 | 139446 |  |
| DW-EC-268 | 139464 | 139661 | OAGBNOCD_00211 hypothetical protein \| JOODPJME_00249 hypothetical protein \| JIHLJMCN_00256 hypothetical protein \| BDMKCPGI_00257 hypothetical protein \| IFPLOHOB_00253 hypothetical protein \| hypothetical protein; *Escherichia* phage phAPEC8 |
| DW-EC-269 | 139753 | 140025 | BDMKCPGI_00258 hypothetical protein \| hypothetical protein phAPEC8_00259; *Escherichia* phage phAPEC8 \| DNHOGCFM_00266 hypothetical protein \| JIHLJMCN_00257 hypothetical protein \| JOODPJME_00248 hypothetical protein \| hypothetical protein; *Escherichia* phage phAPEC8 \| Phage protein]; ACLAME_Phage_proteins_with_unknown_functions Phage_cyanophage  Phage_experimental; *Enterobacteria* phage phi92 \| hypothetical protein phAPEC8_00259; *Escherichia* phage phAPEC8 \| Phi92_gp226; *Enterobacteria* phage phi92 \| hypothetical protein ECGD1_001; *Enterobacteria* phage ECGD1 |
| DW-EC-270 | 140102 | 140359 | JOODPJME_00247 hypothetical protein \| JIHLJMCN_00258 hypothetical protein \| OAGBNOCD_00213 hypothetical protein \| BDMKCPGI_00259 hypothetical protein \| DNHOGCFM_00267 hypothetical protein \| hypothetical protein; *Escherichia* phage phAPEC8 |
| DW-EC-271 | 140361 | 140918 | JOODPJME_00246 hypothetical protein \| JIHLJMCN_00259 hypothetical proteinhfbdacep_00258 hypothetical protein \| BDMKCPGI_00260 hypothetical protein \| DNHOGCFM_00268 hypothetical protein \| hypothetical protein; *Escherichia* phage phAPEC8 |
| DW-EC-272 | 140962 | 141096 | HLAHOEIE_00020 hypothetical protein \| JIHLJMCN_00260 hypothetical protein \| JOODPJME_00245 hypothetical protein \| HFBDACEP_00259 hypothetical protein \| IFPLOHOB_00257 hypothetical protein |
| DW-EC-273 | 141188 | 141550 | JIHLJMCN_00261 hypothetical protein \| JLLHFBGM_00240 hypothetical protein \| OAGBNOCD_00215 hypothetical protein \| BDMKCPGI_00261 hypothetical protein \| IFPLOHOB_00258 hypothetical protein \| hypothetical protein; *Escherichia* phage 2 JES-2013 \| hypothetical protein; *Escherichia* phage vb_ecom_FFH2 \| hypothetical protein; *Escherichia* phage 121Q \| Phage protein; ACLAME_Phage_proteins_with_unknown_functions Phage_cyanophage  Phage_experimental; *Enterobacteria* phage vb_ecom-FV3 \| Phage protein; ACLAME_Phage_proteins_with_unknown_functions Phage_cyanophage  Phage_experimenta; Myoviridae *Escherichia* phage rv5 \| Phi92_gp227 ; *Enterobacteria* phage phi92 \| hypothetical protein Ec2_00173; *Escherichia* phage JES2013 \| hypothetical protein; *Escherichia* phage slur12 \| hypothetical protein; *Escherichia* phage 2 JES-2013; *Escherichia* phage  slur16 \| membrane protein; *Escherichia* phage APCEc0 |
| DW-EC-274 | 141634 | 141813 | JOODPJME_00243 hypothetical protein \| JIHLJMCN_00262 hypothetical protein \| HFBDACEP_00261 hypothetical protein \| IFPLOHOB_00259 hypothetical protein \| hypothetical protein; *Escherichia* phage vb_ecom_FFH2 \| hypothetical protein; *Escherichia* phage 2 JES-2013 \| Phage protein; ACLAME_Phage_proteins_with_unknown_functions Phage_cyanophage Phage_experimental; Myoviridae *Escherichia* phage rv5 \| hypothetical protein CPT_Murica160 ; *Escherichia* phage Murica \| hypothetical protein; *Escherichia* phage V5 \| hypothetical protein ECTP5_00861; *Escherichia* coli O157 typing phage 5 \| hypothetical protein; *Escherichia* phage slur12 \| hypothetical protein Ec2_00165; *Escherichia* phage JES2013 |
| DW-EC-275 | 141911 | 142276 | JOODPJME_00242 hypothetical proteinjihljmcn_00263 hypothetical protein \| IFPLOHOB_00260 hypothetical protein \| OAGBNOCD_00216 hypothetical protein \| HFBDACEP_00262 hypothetical protein \| Phage protein; ACLAME_Phage_proteins_with_unknown_functions Phage_cyanophage Phage_experimental; *Enterobacteria* phage phi92 \| hypothetical protein ECGD1_005; *Enterobacteria* phage ECGD1 \| Phi92_gp231; *Enterobacteria* phage phi92 |
| DW-EC-276 | 142365 | 142604 | JOODPJME_00241 hypothetical protein \| JIHLJMCN_00264 hypothetical protein \| IOGHLCOC_00651 hypothetical proteinioghlcoc_00051 hypothetical protein \| DLFPMBPG_00249 hypothetical protein \| hypothetical protein; *Escherichia* phage 121Q \| hypothetical protein; *Escherichia* phage PBECO 4 \| hypothetical protein PBI_121Q_267; *Escherichia* phage 121Q \| hypothetical protein; *Escherichia* phage PBECO 4 |
| DW-EC-277 | 142640 | 142774 | Phage minor capsid protein, possibly with peptidase activity; ACLAME_Phage_head Phage_Family_Inoviridae Phage_capsid_proteins Phage_coat; Bacteriophage SPP1 \| 7-carboxy-7-deazaguanine synthase; *Rhodopirellula baltica* (strain DSM 10527 / NCIMB 13988 / SH1) |
| DW-EC-278 | 142791 | 142922 | HLAHOEIE_00024 hypothetical proteinifplohob_00262 hypothetical protein \| JOODPJME_00240 hypothetical protein \| JIHLJMCN_00265 hypothetical protein \| OAGBNOCD_00218 hypothetical protein \| hypothetical protein; *Escherichia* phage phAPEC8 \| hypothetical protein phAPEC8_00264; *Escherichia* phage phAPEC8 \| Phi92_gp233; *Enterobacteria* phage phi92 |
| DW-EC-279 | 143013 | 143225 | JOODPJME_00239 hypothetical protein \| JIHLJMCN_00266 hypothetical protein \| IFPLOHOB_00263 hypothetical protein \| ELONAMNK_00012 hypothetical protein \| OLDBFMDK_00105 hypothetical protein \| phosphoribosylpyrophosphate synthetase; *Escherichia* phage Av-05 \| hypothetical protein; *Escherichia* phage 121Q |
| DW-EC-280 | 143232 | 143321 |  |
| DW-EC-281 | 143318 | 143485 | HLAHOEIE_00026 hypothetical protein \| JOODPJME_00238 hypothetical protein \| JIHLJMCN_00267 hypothetical protein \| HFBDACEP_00266 hypothetical protein \| DNHOGCFM_00273 hypothetical protein \| hypothetical protein; *Escherichia* phage phAPEC8 \| hypothetical protein phAPEC8_00266; *Escherichia* phage phAPEC8 \| hypothetical protein ECGD1_008; *Enterobacteria* phage ECGD1 \| Phi92_gp235; *Enterobacteria* phage phi92 |
| DW-EC-282 | 143560 | 143781 | IFPLOHOB_00266 hypothetical protein \| OAGBNOCD_00221 hypothetical protein \| HFBDACEP_00267 hypothetical protein \| DNHOGCFM_00274 hypothetical protein \| hypothetical protein phAPEC8_00267; *Escherichia* phage phAPEC8 \| hypothetical protein; *Escherichia* phage phAPEC8 |
| DW-EC-283 | 143861 | 144214 | JOODPJME_00235 hypothetical protein \| JIHLJMCN_00270 hypothetical protein \| OAGBNOCD_00222 hypothetical protein \| HLAHOEIE_00028 hypothetical protein \| HFBDACEP_00268 hypothetical protein \| hypothetical protein; *Escherichia* phage phAPEC8 \| hypothetical protein phAPEC8_00268; *Escherichia* phage phAPEC8 |
| DW-EC-284 | 144294 | 144440 | JOODPJME_00234 hypothetical protein \| JIHLJMCN_00271 hypothetical protein \| OAGBNOCD_00223 hypothetical protein \| HLAHOEIE_00029 hypothetical protein \| HFBDACEP_00269 hypothetical protein \| hypothetical protein; *Escherichia* phage phAPEC8 \| hypothetical protein phAPEC8_00269; *Escherichia* phage phAPEC8 |
| DW-EC-285 | 144496 | 144681 | OAGBNOCD_00224 hypothetical protein \| HFBDACEP_00270 hypothetical protein \| JOODPJME_00233 hypothetical protein \| JIHLJMCN_00272 hypothetical protein \| IFPLOHOB_00269 hypothetical protein \| hypothetical protein; *Escherichia* phage phAPEC8 \| Cysteine protease ATG4C; Xenopus tropicalis \| Cysteine protease ATG4C; Xenopus laevis |
| DW-EC-286 | 144767 | 144973 | OAGBNOCD_00225 hypothetical protein \| HLAHOEIE_00031 hypothetical protein \| hypothetical protein; *Escherichia* phage phAPEC8 |
| DW-EC-287 | 145015 | 145329 | DNHOGCFM_00003 hypothetical protein \| HFBDACEP_00272 hypothetical proteinjoodpjme_00231 hypothetical protein \| IFPLOHOB_00271 hypothetical protein \| JIHLJMCN_00274 hypothetical protein \| hypothetical protein; *Escherichia* phage phAPEC8 |
| DW-EC-288 | 145427 | 145546 | HLAHOEIE_00033 hypothetical protein \| HFBDACEP_00273 hypothetical protein \| IFPLOHOB_00272 hypothetical protein \| DNHOGCFM_00004 hypothetical protein \| hypothetical protein phAPEC8_004; *Escherichia* phage phAPEC8 \| hypothetical protein; *Escherichia* phage phAPEC8 |
| DW-EC-289 | 145557 | 145718 | JIHLJMCN_00276 hypothetical protein \| OAGBNOCD_00227 hypothetical protein \| HLAHOEIE_00034 hypothetical protein \| HFBDACEP_00274 hypothetical protein \| IFPLOHOB_00273 hypothetical protein \| hypothetical protein; *Escherichia* phage phAPEC8 |
| DW-EC-290 | 145728 | 145838 | OAGBNOCD_00228 hypothetical protein \| hypothetical protein phAPEC8_007; *Escherichia* phage phAPEC8 \| hypothetical protein; *Escherichia* phage phAPEC8 |
| DW-EC-291 | 145866 | 146201 | JOODPJME_00229 hypothetical protein \| JIHLJMCN_00277 hypothetical protein \| DCKEIKPP_00012 hypothetical protein \| JLLHFBGM_00253 hypothetical protein \| OAGBNOCD_00229 hypothetical protein \| hypothetical protein; *Escherichia* phage phAPEC8 \| Phage protein; ACLAME_Phage_proteins_with_unknown_functions Phage_cyanophage Phage_experimental; *Enterobacteria* phage phi92 \| hypothetical protein phAPEC8_008; *Escherichia* phage phAPEC8 \| Phi92_gp242; *Enterobacteria* phage phi92 |
| DW-EC-292 | 146287 | 146457 | DNHOGCFM_00008 hypothetical protein \| JOODPJME_00228 hypothetical protein \| ELONAMNK_00004 hypothetical protein \| HLAHOEIE_00036 hypothetical protein \| OLDBFMDK_00097 hypothetical protein \| hypothetical protein; *Escherichia* phage phAPEC8 |
| DW-EC-293 | 146502 | 146639 | JOODPJME_00227 hypothetical protein \| JIHLJMCN_00279 hypothetical protein \| IFPLOHOB_00276 hypothetical protein \| NNKEDCCG_00007 hypothetical protein \| MILOOAOH_00212 hypothetical protein |
| DW-EC-294 | 146723 | 146827 | DNHOGCFM_00009 hypothetical protein \| NNKEDCCG_00006 hypothetical protein \| PKGENPCH_00138 hypothetical protein \| MILOOAOH_00213 hypothetical protein \| NLAEJCGN_00007 hypothetical protein \| Phage protein; ACLAME_Phage_proteins_with_unknown_functions Phage_cyanophage Phage_experimental; *Enterobacteria* phage vb_ecom-FV3 |
| DW-EC-295 | 146838 | 147053 | JIHLJMCN_00280 hypothetical protein \| HLAHOEIE_00038 hypothetical protein \| HFBDACEP_00278 hypothetical protein \| DNHOGCFM_00010 hypothetical protein \| IFPLOHOB_00278 hypothetical protein \| hypothetical protein; *Escherichia* phage phAPEC8 \| hypothetical protein phAPEC8_0010; *Escherichia* phage phAPEC8 \| hypothetical protein apcec02_060; *Escherichia* phage apcec02 |
| DW-EC-296 | 147050 | 147436 | BDMKCPGI_00267 hypothetical protein \| DNHOGCFM_00011 hypothetical protein \| hypothetical protein phAPEC8_0011; *Escherichia* phage phAPEC8 \| HLAHOEIE_00039 hypothetical protein \| DCKEIKPP_00008 hypothetical protein \| hypothetical protein; *Escherichia* phage phAPEC8 \| hypothetical protein phAPEC8_0011; *Escherichia* phage phAPEC8 |
| DW-EC-297 | 147520 | 147711 | HLAHOEIE_00040 hypothetical protein \| IFPLOHOB_00280 hypothetical protein \| OAGBNOCD_00234 hypothetical protein |
| DW-EC-298 | 147790 | 148089 | JOODPJME_00224 hypothetical protein \| JIHLJMCN_00282 hypothetical protein \| DCKEIKPP_00007 hypothetical protein \| HLAHOEIE_00041 hypothetical protein \| BDMKCPGI_00268 hypothetical protein \| hypothetical protein; *Escherichia* phage phAPEC8 |
| DW-EC-299 | 148134 | 148370 | PINPCHNM_00249 hypothetical protein \| Phi92_gp244; *Enterobacteria* phage phi92 \| DNHOGCFM_00013 hypothetical protein \| hypothetical protein phAPEC8_0013; *Escherichia* phage phAPEC8 \| JOODPJME_00223 hypothetical protein \| Phage protein; ACLAME_Phage_proteins_with_unknown_functions Phage_cyanophage Phage_experimental; *Enterobacteria* phage phi92 \| hypothetical protein; *Escherichia* phage phAPEC8 |
| DW-EC-300 | 148426 | 148710 | DCKEIKPP_00005 hypothetical protein \| DNHOGCFM_00014 hypothetical protein \| hypothetical protein phAPEC8_0014; *Escherichia* phage phAPEC8 \| HLAHOEIE_00042 hypothetical protein \| OAGBNOCD_00236 hypothetical protein \| hypothetical protein; *Escherichia* phage phAPEC8 \| hypothetical protein; *Escherichia* phage vb_ecom_FFH2 |
| DW-EC-301 | 148713 | 148847 | HLAHOEIE_00043 hypothetical protein \| OAGBNOCD_00237 hypothetical protein \| hypothetical protein; *Escherichia* phage 2 JES-2013 \| hypothetical protein; *Escherichia* phage phAPEC8 |
| DW-EC-302 | 148864 | 148962 | Phage terminase, small subunit; ACLAME_Phage_head Phage_packaging_machinery T4-like_phage_core_proteins zzRobE_test; Caudovirales *Geobacillus* phage GBSV1 \| Phage terminase, small subunit; ACLAME_Phage_head Phage_packaging_machinery T4-like_phage_core_proteins zzRobE_test; Caudovirales *Bacillus* virus 1 \| hypothetical protein BV1_gp17; *Bacillus* virus 1 \| hypothetical protein GPGV1_gp16; *Geobacillus* phage GBSV1 \| beta-galactosidase (EC 3.2.1.23) |
| DW-EC-303 | 149100 | 149333 | HFBDACEP_00283 hypothetical protein \| JIHLJMCN_00285 hypothetical protein \| HLAHOEIE_00044 hypothetical protein \| BDMKCPGI_00271 hypothetical protein \| IFPLOHOB_00286 hypothetical protein \| hypothetical protein; *Escherichia* phage 121Q \| Phage protein; ACLAME_Phage_proteins_with_unknown_functions Phage_cyanophage  Phage_experimental; *Enterobacteria* phage phi92 \| hypothetical protein PBI_121Q_272; *Escherichia* phage 121Q \| Phi92_gp247; *Enterobacteria* phage phi92 |
| DW-EC-304 | 149412 | 149780 | JIHLJMCN_00286 hypothetical protein \| DNHOGCFM_00015 hypothetical protein \| hypothetical protein phAPEC8_001; *Escherichia* phage phAPEC8 \| HLAHOEIE_00045 hypothetical protein \| HFBDACEP_00284 hypothetical protein \| hypothetical protein; *Escherichia* phage phAPEC8 |
| DW-EC-305 | 149832 | 149924 | Hypothetical protein; *Bacillus* phage Spock \| hypothetical protein Spock_150; *Bacillus* phage Spock |
| DW-EC-306 | 149921 | 150340 | HLAHOEIE_00046 hypothetical proteindnhogcfm_00016 hypothetical protein \| hypothetical protein phAPEC8_0017; *Escherichia* phage phAPEC8 \| JOODPJME_00218 hypothetical protein \| JIHLJMCN_00287 hypothetical protein \| hypothetical protein; *Escherichia* phage phAPEC8 \| Phage protein; ACLAME_Phage_proteins_with_unknown_functions Phage_cyanophage Phage_experimental; Myoviridae *Escherichia* phage rv5 \| hypothetical protein; *Escherichia* phage vb_ecom_FFH2 \| hypothetical protein; *Escherichia* phage 2 JES-2013 |
| DW-EC-307 | 150335 | 150463 | Virion structural protein; *Synechococcus* phage S-mbcm6 \| neck protein; *Synechococcus* phage S-mbcm25 \| virion structural protein; *Synechococcus* phage ACG-2014c |
| DW-EC-308 | 150467 | 150565 |  |
| DW-EC-309 | 150641 | 150751 | Beta-galactosidase 15; *Arabidopsis thaliana* \| beta-galactosidase (EC 3.2.1.23) \| exo-beta-glucosaminidase (EC 3.2.1.165) \| exo-beta-1,4-galactanase (EC 3.2.1.-) \| beta-1,3-galactosidase (EC 3.2.1.-) |
| DW-EC-310 | 150752 | 150889 |  |
| DW-EC-311 | 151036 | 151134 |  |
| DW-EC-312 | 151118 | 151264 |  |
| DW-EC-313 | 151429 | 151578 |  |
| DW-EC-314 | 151667 | 151876 |  |
| DW-EC-315 | 55043 | 55117 | tRNA-Ile2(CAT) |
| DW-EC-316 | 55120 | 55194 | tRNA-Arg(TCT) |
| DW-EC-317 | 55429 | 55519 | tRNA-Ser(TGA) |
| DW-EC-318 | 55525 | 55610 | tRNA-Ser(GCT) |
| DW-EC-319 | 55741 | 55828 | tRNA-Tyr(GTA) |
| DW-EC-320 | 55835 | 55920 | tRNA-Asn(GTT) |
| DW-EC-321 | 55928 | 56004 | tRNA-Undet(NNN) |
| DW-EC-322 | 56011 | 56085 | tRNA-Thr(TGT) |
| DW-EC-323 | 56400 | 56473 | tRNA-Gly(TCC) |
| DW-EC-324 | 56572 | 56647 | tRNA-Gln(TTG) |
| DW-EC-325 | 56653 | 56729 | tRNA-Pro(TGG) |
| DW-EC-326 | 56736 | 56809 | tRNA-Ile(GAT) |
| DW-EC-327 | 56888 | 56966 | tRNA-fmet(CAT) |

Supplementary Table S5 DW-EC Application on Various Food at 4 ^o^C Storage Temperature

| **Samples** | **Incubation Time (Day)** | **Control (CFU/mL)** | **Bacteriophage Treatment (CFU/mL)** | **Bacterial Reduction (log_10_)** | **Bacterial**  **Reduction (%)** |
| --- | --- | --- | --- | --- | --- |
| Chicken | 1 | 1.24 ± 2.07 x 10^4i^ | 2.37 ± 0.87 x 10^3d*^ | 0.72 ± 0.01 | 80.93 ± 0.51 |
|  | 6 | 3.16 ± 1.35 x 10^3cd^ | 4.03 ± 1.11 x 10^2a*^ | 0.90 ± 0.03 | 87.29 ± 0.88 |
| Fish | 1 | 8.59 ± 0.99 x 10^3h^ | 3.11 ± 0.85 x 10^3f*^ | 0.44 ± 0.001 | 63.78 ± 0.09 |
|  | 6 | 4.32 ± 1.78 x 10^3e^ | 5.17 ± 0.78 x 10^2ab^ | 0.92 ± 0.02 | 87.89 ± 0.64 |
| Cucumber | 1 | 7.44 ± 1.50 x 10^3g^ | 2.87 ± 0.75 x 10^3e*^ | 0.41 ± 0.003 | 61.42 ± 0.25 |
|  | 6 | 2.30 ± 1.69 x 10^3bc^ | 6.50 ± 0.77 x 10^2b*^ | 0.55 ± 0.02 | 71.88 ± 1.00 |
| Tomato | 1 | 3.38 ± 1.02 x 10^3d^ | 1.48 ± 1.01 x 10^3c*^ | 0.48 ± 0.13 | 56.24 ± 1.14 |
|  | 6 | 2.01 ± 1.17 x 10^3b^ | 5.12 ± 0.57 x 10^2ab*^ | 0.59 ± 0.01 | 74.51 ± 0.82 |
| Lettuce | 1 | 6.64 ± 1.09 x 10^3f^ | 3.42 ± 1.16 x 10^3g*^ | 0.28 ± 0.01 | 46.88 ± 1.50 |
|  | 6 | 7.47 ± 1.39 x 10^2a^ | 4.23 ± 0.82 x 10^2a*^ | 0.25 ± 0.004 | 43.38 ± 0.58 |

Data were shown in mean ± standard error value, different letters in each column indicated significant differences, α ≤ 0.05. “*”: shown significant differences between control and bacteriophage treatment for each sample.
